# The different clinical facets of *SYN1*-related neurodevelopmental disorders

**DOI:** 10.3389/fcell.2022.1019715

**Published:** 2022-12-08

**Authors:** Ilaria Parenti, Elsa Leitão, Alma Kuechler, Laurent Villard, Cyril Goizet, Cécile Courdier, Allan Bayat, Alessandra Rossi, Sophie Julia, Ange-Line Bruel, Frédéric Tran Mau-Them, Sophie Nambot, Daphné Lehalle, Marjolaine Willems, James Lespinasse, Jamal Ghoumid, Roseline Caumes, Thomas Smol, Salima El Chehadeh, Elise Schaefer, Marie-Thérèse Abi-Warde, Boris Keren, Alexandra Afenjar, Anne-Claude Tabet, Jonathan Levy, Anna Maruani, Ángel Aledo-Serrano, Waltraud Garming, Clara Milleret-Pignot, Anna Chassevent, Marije Koopmans, Nienke E. Verbeek, Richard Person, Rebecca Belles, Gary Bellus, Bonnie A. Salbert, Frank J. Kaiser, Laure Mazzola, Philippe Convers, Laurine Perrin, Amélie Piton, Gert Wiegand, Andrea Accogli, Francesco Brancati, Fabio Benfenati, Nicolas Chatron, David Lewis-Smith, Rhys H. Thomas, Federico Zara, Pasquale Striano, Gaetan Lesca, Christel Depienne

**Affiliations:** ^1^ Institute of Human Genetics, University Hospital Essen, University Duisburg-Essen, Essen, Germany; ^2^ INSERM, MMG, Faculté de Médecine, Aix-Marseille University, Marseille, France; ^3^ Département de Génétique Médicale, APHM, Hôpital d'Enfants de La Timone, Marseille, France; ^4^ Service de Génétique Médicale, Bordeaux, France; ^5^ Centre de Référence Maladies Rares Neurogénétique, Service de Génétique Médicale, Bordeaux, France; ^6^ NRGEN Team, INCIA, CNRS UMR 5287, University of Bordeaux, Bordeaux, France; ^7^ Institute for Regional Health Services, University of Southern Denmark, Odense, Denmark; ^8^ Department of Epilepsy Genetics and Personalized Medicine, Danish Epilepsy Center, Dianalund, Denmark; ^9^ Department of Drug Design and Pharmacology, University of Copenhagen, Copenhagen, Denmark; ^10^ Pediatric Clinic, IRCCS Policlinico San Matteo Foundation, University of Pavia, Pavia, Italy; ^11^ Service de Génétique Médicale, Pôle de Biologie, CHU de Toulouse - Hôpital Purpan, Toulouse, France; ^12^ Unité Fonctionnelle Innovation en Diagnostic Génomique des Maladies Rares, FHU-TRANSLAD, CHU Dijon Bourgogne, Dijon, France; ^13^ UMR1231 GAD, Inserm - Université Bourgogne-Franche Comté, Dijon, France; ^14^ Department of Medical Genetics, Rare diseases and Personalized Medicine, CHU Montpellier, University of Montpellier, Montpellier, France; ^15^ Inserm U1298, INM, CHU Montpellier, University of Montpellier, Montpellier, France; ^16^ Service de Cytogenetique, Centre Hospitalier de Chambéry, Chambéry, France; ^17^ Univ. Lille, ULR7364 RADEME, Lille, France; ^18^ CHU Lille, Clinique de Génétique, Guy Fontaine, Lille, France; ^19^ CHU Lille, Institut de Génétique Médicale, Lille, France; ^20^ Service de Génétique Médicale, Institut de Génétique Médicale d'Alsace (IGMA), Hôpitaux Universitaires de Strasbourg, Hôpital de Hautepierre, Strasbourg, France; ^21^ Département de NeuroPédiatrie, Hôpitaux Universitaires de Strasbourg, Strasbourg, France; ^22^ APHP, Département de Génétique, UF de Génomique du Développement, Département de Génétique, Groupe Hospitalier Pitié-Salpêtrière, Sorbonne Université, Paris, France; ^23^ Département de Génétique, Centre de Référence déficiences Intellectuelles de Causes Rares, APHP, Hôpital Armand Trousseau, Sorbonne Université, Paris, France; ^24^ APHP, Département de Génétique, Hôpital Robert-Debré, Paris, France; ^25^ Department of Child and Adolescent Psychiatry, Robert Debré Hospital, APHP, Paris, France; ^26^ Epilepsy and Neurogenetics Program, Neurology Department, Ruber Internacional Hospital, Madrid, Spain; ^27^ Sozialpädiatrisches Zentrum, Kinder-und Jugendklinik Gelsenkirchen, Gelsenkirchen, Germany; ^28^ Service de Pédiatrie, CH de Mâcon, Mâcon, France; ^29^ Department of Neurogenetics, Kennedy Krieger Institute, Baltimore, MD, United States; ^30^ Department of Genetics, Utrecht University Medical Center, Utrecht, Netherlands; ^31^ GeneDx, Gaithersburg, MD, United States; ^32^ Medical Genetics, Geisinger Medical Center, Danville, PA, United States; ^33^ Essener Zentrum für Seltene Erkrankungen (EZSE), Universitätsklinikum Essen, Essen, Germany; ^34^ Department of Neurology, University Hospital, Lyon Neuroscience Research Center (CRNL), INSERM U1028, CNRS UMR5292, Lyon, France; ^35^ Department of Neurology, University Hospital, Saint-Etienne, France; ^36^ Department of Paediatric Physical Medicine and Rehabilitation, CHU Saint-Étienne, Hôpital Bellevue, Rhône-Alpes Reference Centre for Neuromuscular Diseases, Saint-Étienne, France; ^37^ Institut de Génétique et de Biologie Moléculaire et Cellulaire, Illkirch, France; ^38^ Centre National de la Recherche Scientifique, UMR7104, Illkirch, France; ^39^ Institut National de la Santé et de la Recherche Médicale, U964, Illkirch, France; ^40^ Université de Strasbourg, Illkirch, France; ^41^ Division of Pediatric Neurology, Department of Pediatrics, Asklepios Klinik Nord-Heidberg, Hamburg, Germany; ^42^ Department of Pediatric and Adolescent Medicine II (Neuropediatrics, Social Pediatrics), University Medical Centre Schleswig-Holstein, Kiel, Germany; ^43^ Department of Specialized Medicine, Division of Medical Genetics, McGill University Health Centre, Montreal, Qc, Canada; ^44^ Department of Human Genetics, Faculty of Medicine, McGill University, Montreal, Qc, Canada; ^45^ Department of Life, Human Genetics, Health and Environmental Sciences, University of L’Aquila, L’Aquila, Italy; ^46^ IRCCS San Raffaele Roma, Rome, Italy; ^47^ Center for Synaptic Neuroscience and Technology, Istituto Italiano di Tecnologia, Geneva, Italy; ^48^ IRCCS Ospedale Policlinico San Martino, Geneva, Italy; ^49^ Service de Genetique, Hospices Civils de Lyon, Bron, France; ^50^ Institute NeuroMyoGène, Laboratoire Physiopathologie et Génétique du Neurone et du Muscle, CNRS UMR 5261 -INSERM U1315, Université de Lyon - Université Claude Bernard Lyon 1, Lyon, France; ^51^ Translational and Clinical Research Institute, Newcastle University, Newcastle Upon Tyne, United Kingdom; ^52^ Department of Clinical Neurosciences, Newcastle Upon Tyne Hospitals NHS Foundation Trust, Newcastle Upon Tyne, United Kingdom; ^53^ IRCCS G. Gaslini, Genova, Italy; ^54^ Department of Neurology, Rehabilitation, Ophtalmology, Genetics, Maternal and Child Health, University of Genova, Genova, Italy

**Keywords:** SYN1, synapsins, reflex epilepsy, genotype-phenotype correlation, neurodevelopmental disorders, autism spectrum disorders

## Abstract

Synapsin-I (SYN1) is a presynaptic phosphoprotein crucial for synaptogenesis and synaptic plasticity. Pathogenic *SYN1* variants are associated with variable X-linked neurodevelopmental disorders mainly affecting males. In this study, we expand on the clinical and molecular spectrum of the *SYN1*-related neurodevelopmental disorders by describing 31 novel individuals harboring 22 different *SYN1* variants. We analyzed newly identified as well as previously reported individuals in order to define the frequency of key features associated with these disorders. Specifically, behavioral disturbances such as autism spectrum disorder or attention deficit hyperactivity disorder are observed in 91% of the individuals, epilepsy in 82%, intellectual disability in 77%, and developmental delay in 70%. Seizure types mainly include tonic-clonic or focal seizures with impaired awareness. The presence of reflex seizures is one of the most representative clinical manifestations related to *SYN1*. In more than half of the cases, seizures are triggered by contact with water, but other triggers are also frequently reported, including rubbing with a towel, fever, toothbrushing, fingernail clipping, falling asleep, and watching others showering or bathing. We additionally describe hyperpnea, emotion, lighting, using a stroboscope, digestive troubles, and defecation as possible triggers in individuals with *SYN1* variants. The molecular spectrum of *SYN1* variants is broad and encompasses truncating variants (frameshift, nonsense, splicing and start-loss variants) as well as non-truncating variants (missense substitutions and in-frame duplications). Genotype-phenotype correlation revealed that epileptic phenotypes are enriched in individuals with truncating variants. Furthermore, we could show for the first time that individuals with early seizures onset tend to present with severe-to-profound intellectual disability, hence highlighting the existence of an association between early seizure onset and more severe impairment of cognitive functions. Altogether, we present a detailed clinical description of the largest series of individuals with *SYN1* variants reported so far and provide the first genotype-phenotype correlations for this gene. A timely molecular diagnosis and genetic counseling are cardinal for appropriate patient management and treatment.

## Introduction

Synapsins are a family of presynaptic phosphoproteins mainly expressed in the central and peripheral nervous system (Ueda and Greengard 1977; De Camilli, Cameron, and Greengard 1983). They play a fundamental role during the early stages of neuronal development, where they regulate neurite outgrowth, synaptic development, function, and plasticity through the regulation of the number of synaptic vesicles (SVs) available for neurotransmitter release (Cesca et al. 2010).

Synapsins exist in both invertebrates and vertebrates. Mammals possess three synapsin genes named *SYN1*, *SYN2* and *SYN3. SYN1* is located on chromosome X, *SYN2* and *SYN3* are on autosomes. *SYN1* and *SYN2* each produce two different protein isoforms through alternative splicing (SYN1a, SYN1b, SYN2a, SYN2b). The different isoforms share a common N-terminal region and diverge at the C-terminus (Hosaka and Sudhof 1998; Sudhof et al. 1989). The N-terminus of synapsins is composed of domains A-C. Domain A comprises residues that are target of phosphorylation and therefore important for the regulation of the activity of the protein. Domain B is relatively poorly conserved and is considered a linker between domains A and C. The last N-terminal domain, namely domain C, is composed of 300 amino acids and is essential for the activity of the protein. The domains of the C-terminal region (D-J) are instead highly variable and differ between both the primary transcripts and the splice variants (Longhena et al. 2021; Cesca et al. 2010). Synapsin protein isoforms display partially overlapping functions but are also characterized by distinctive localization and expression patterns, as well as different post-translational modifications (Guarnieri et al. 2017; Longhena et al. 2021). The finely orchestrated balance between the isoforms is essential for proper brain functions. Importantly, the vast majority of neurons within the central nervous system express at least one synapsin isoform (De Camilli, Cameron, and Greengard 1983).

Alteration of synapsin function results in neurodevelopmental phenotypes in both humans and mice. In mice, the disruption of a single synapsin gene has only a mild impact on learning and behavior, while double or triple gene knockouts display additive effects which lead to more severe phenotypes (Etholm and Heggelund 2009; Cambiaghi et al. 2013). Late-onset seizures triggered by sensitive stimuli occur in *Syn1* ^−/−^, *Syn2* ^−/−^, as well as in *Syn1/Syn2* and *Syn1/Syn2/Syn3* double and triple knockout mice, but not in *Syn3* ^−/−^ mice (Corradi et al. 2008; Etholm et al. 2012; Rosahl et al. 1995; Gitler et al. 2004; Feng et al. 2002). In humans, variants in *SYN1* have been independently associated with epilepsy, learning disabilities and behavioral disorders (OMIM #300491) (Garcia et al. 2004; Fassio et al. 2011) or non-syndromic intellectual disability (OMIM #300115) (Guarnieri et al. 2017). In mice, alterations of *Syn2*, and to a lesser extent of *Syn1*, are associated with autistic-like features and rare seizures (Etholm et al. 2012; Greco et al. 2013; Michetti et al. 2017). Autistic behavioral features seem to predominate in mice and human individuals with *syn2/SYN2* variants, whereas focal-onset seizures seem more characteristic of *syn1/SYN1* (Etholm et al. 2012). The evidence for an association between rare variants in *SYN2* and epilepsy remains weak as the missense variants carried by the single described case has subsequently been reported in gnomAD (Corradi et al. 2014). Early suggestions of an association between epilepsy and a common intron variant in *SYN2* (Cavalleri et al. 2007) have become less compelling in larger genome-wide association studies (International League Against Epilepsy Consortium on Complex, 2018). Finally, disruption of *Syn*3 also alters social behaviors in mice (Michetti et al. 2017; Greco et al. 2013) but a clear association between *SYN3* and human disease has not yet been established, although *de novo* variants of unknown significance in this gene have been reported in two patients with developmental disorders (Eldomery et al. 2017; Turner et al. 2019).

Here, we focus on the X-linked gene *SYN1.* Pathogenic variants in this gene have been recurrently reported in individual cases and families (Garcia et al. 2004; Fassio et al. 2011; Guarnieri et al. 2017; Xiong et al. 2021; Zhou et al. 2021; Butler et al. 2017; Lindy et al. 2018; Nguyen et al. 2015; Rossi et al. 2017; Peron et al. 2018; Fernandez-Marmiesse et al. 2019; Darvish et al. 2020; Ibarluzea et al. 2020; Mojarad et al. 2021; van der Ven et al. 2021; Stranneheim et al. 2021; Yang et al. 2020; Cabana et al. 2018). Epilepsy, learning disabilities, speech delay, intellectual disability (ID), and autism spectrum disorder (ASD) are the most frequent clinical manifestations associated with *SYN1* variants (Longhena et al. 2021; John et al. 2021; Accogli et al. 2021), but an extensive comparison of the clinical features and genotype-phenotype correlations are missing. Recent studies have highlighted a specific association of reflex epilepsy with *SYN1* variants (Nguyen et al. 2015; Peron et al. 2018; Accogli et al. 2021), but the percentage of patients exhibiting this phenotype and the full clinical spectrum associated with *SYN1* remains undetermined. Reflex epilepsy can be defined as the reproducible provocation of seizures in response to a specific motor, sensory, or cognitive stimulus, with or without the occurrence of spontaneous seizures. In patients with *SYN1* variants, seizures are frequently triggered by bathing or showering. Importantly, *SYN1*-related disorders are characterized by extensive clinical heterogeneity and intrafamilial variability.

Herein, we report 31 unpublished individuals from 22 families harboring variants in *SYN1* and review variants and clinical features published in the literature in order to deep-phenotype *SYN1*-related disorders and explore potential genotype-phenotype correlations.

## Materials and methods

### *SYN1* study cohort

Our cohort was collected through an International collaboration (EuroEPINOMICS RES consortium) and GeneMatcher (Sobreira et al. 2015). Gene panels and exome sequencing were performed at the respective institutions. All procedures were performed following the ethical standards of the institutional and/or national research committee, and in conformity with the 1964 Helsinki declaration and its later amendments or comparable ethical standards. After anonymization of patients’ data, each referring physician provided detailed developmental, neurological, and behavioral history of the patients. Informed consent was obtained from all individuals included in this study or their legal guardians. Clinical features of the previously published cases were retrieved from the corresponding publications.

### *SYN1* variants

Variants were mapped on the *SYN1* NM_006950.3 RefSeq transcript using HGVS recommendations (den Dunnen et al. 2016) and classified according to ACMG Guidelines (Richards et al. 2015). All variants have been submitted to the ClinVar Database and have been assigned the following accession numbers: SCV002558865 - SCV002558886.

Known *SYN1* ENST00000295987.7 (corresponding to NM_006950.3) variants were retrieved from gnomAD v2.1.1 (Karczewski et al. 2020), restricting to loss-of-function, missense and synonymous single nucleotide variants or indels. For each gnomAD variant, we calculated the number of females and males carrying the variant. The combined annotation-dependent depletion (CADD) score (Rentzsch et al. 2019) (https://cadd.gs.washington.edu/score) was calculated for all variants using GRCh37-v1.6 genomic coordinates.

Putative effects of the variants on splicing were calculated with the SpliceAI neural network (Jaganathan et al. 2019). The probability that a variant affects splicing is expressed as score cutoffs, namely >0.5 (recommended) and >0.8 (high precision).

Pathogenic and likely pathogenic *SYN1* variants were retrieved from HGMD Professional (Qiagen, Hilden, Germany). Three variants reported in the HGMD database were excluded from the total count: p.(Ala51Gly) and p.(Thr567Ala) were previously deemed benign due to minor allele frequency (MAF) thresholds and computational evidence (Abouelhoda et al. 2016; Thauvin-Robinet et al. 2019), whereas p.(Gly240Arg) shows an incongruent pattern of inheritance (Fernandez-Marmiesse et al. 2019).

### *In silico* SYN1 protein analysis

Amino acid positions of each protein region of SYN1 were retrieved from Uniprot (The UniProt Consortium et al., 2021). Evolutionary conservation of the SYN1 amino acids affected by missense substitutions was retrieved from Alamut Visual Plus™ (SOPHiA GENETICS, Lausanne, Switzerland). Alignment of amino acids across synapsins was carried out with Clustal Omega (Goujon et al. 2010; Sievers et al. 2011).

The putative effects of the missense substitutions on protein stability were predicted with DynaMut2 (Rodrigues, Pires, and Ascher 2021) and mCSM (Pires, Ascher, and Blundell 2014). The AlphaFold SYN1 protein structure (AF-P17600-F1-model_v3) was used for the analysis. Residues with a confidence score <70 based on AlphaFold predictions were excluded from the analysis.

### Statistics

Fisher’s tests were performed to determine associations 1) between truncating variants and the manifestation of each main clinical feature, 2) between sex and the manifestation of the clinical features of interest, and 3) between sex and the existence of putatively damaging variants using gnomAD data. Odds ratios were log2 transformed and indicate enrichment or depletion of genes, for positive or negative values, respectively. *p*-values were adjusted for multiple comparisons using Bonferroni correction.

Mann-Whitney tests followed by Bonferroni correction for multiple testing were used to compare the age of epilepsy onset between individuals with different levels of ID.

## Results

### Classification of *SYN1* variants

**FIGURE 1:**
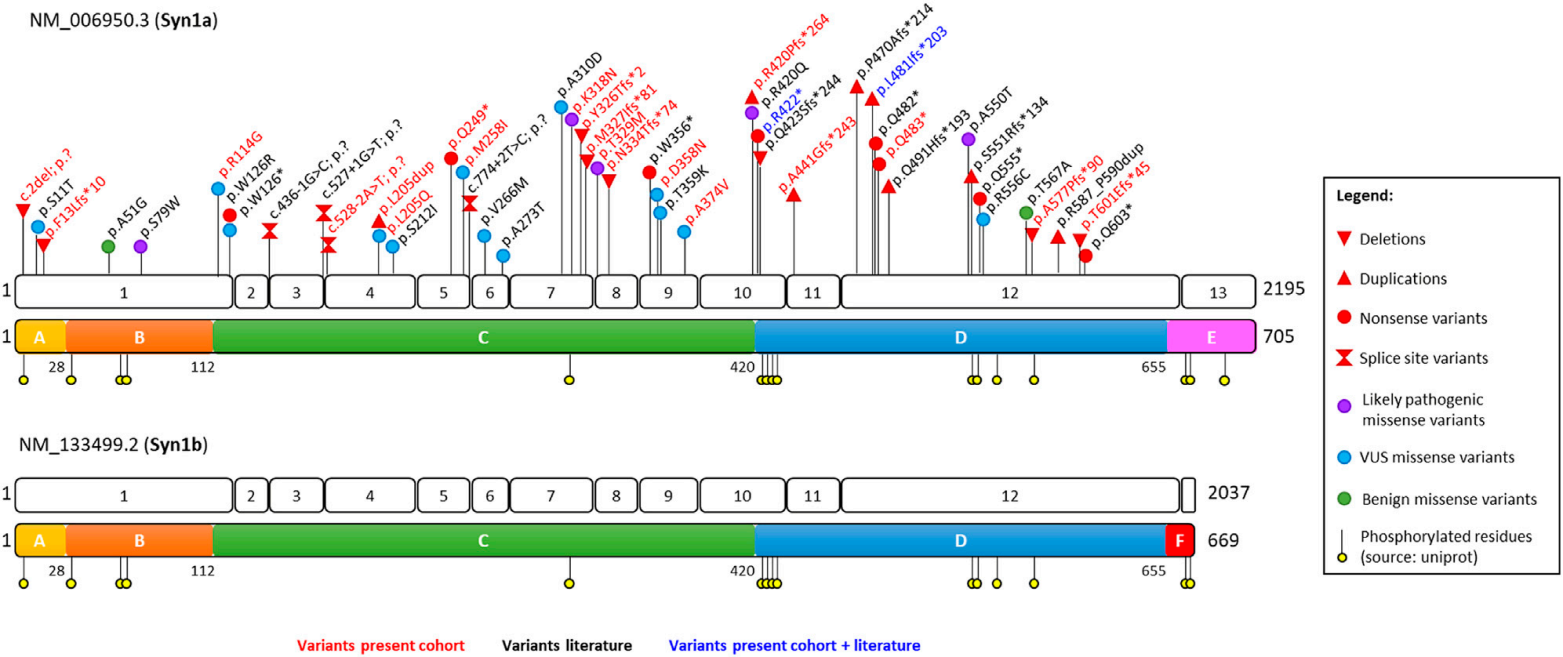
Schematic representation of the *SYN1* gene and SYN1 protein with positions of the identified variants relative to exon and domains distribution. Variants identified in the present cohort are depicted in red, those reported in the literature in black, and those reported both in the literature and in our cohort in blue. The conserved A (N-terminal highly phosphorylated domain), B (linker), and C (functional domain) domains are depicted in yellow, orange, and green, respectively; the variable D, E, and F domains in blue, pink, and red. Classification of the variants was performed based on ACMG criteria. Missense substitutions are subdivided in likely pathogenic, VUS, and benign based on the presence of functional data and their frequency in the healthy population.

Twenty-two different genetic alterations of *SYN1* were identified in a total of 31 individuals. Detailed information about each variant is available in Supplementary Table S1. The identified variants comprise nine frameshift variants, three nonsense variants, one start-loss, one splice site variant, seven missense substitutions, and one in-frame duplication. The newly identified variants almost double the total number of reported *SYN1* alterations, raising it to 46 (13 frameshift, eight nonsense, 18 missense, four altering splice sites, one start-loss and two in-frame duplications). All variants reported so far are indicated on the corresponding transcript and protein domain in Figure 1. As shown in this Figure, all variants alter amino acids shared by both SYN1 isoforms (SYN1a and SYN1b), while the variable C-terminal domains E and F are spared. No apparent clustering of variants of unknown significance (VUS) or likely pathogenic missense substitutions can be observed.

CADD scores were calculated for all variants and range from 20.3 to 40. As expected, truncating variants are associated with higher CADD scores, whereas the two in-frame duplications show the lowest scores. Variants were additionally classified based on ACMG guidelines. Of the 46 variants, 26 were classified as pathogenic, five as likely pathogenic and 15 as variants of unknown significance (VUS) (Supplementary Table S1). Missense substitutions and in-frame duplications fall within the category of VUS, unless functional studies (PS3) or a *de novo* origin with confirmed maternity and paternity (PS2) are reported.

Likely pathogenic and VUS missense substitutions were evaluated based on the conservation of the affected amino acids across species and synapsin isoforms (Supplementary Figures S1, 2). Except for p.(Arg114Gly), all missense substitutions affect evolutionarily conserved amino acids (Supplementary Figure S1). Arginine 114 also differs among SYN1, SYN2, and SYN3. Eleven missense variants (p.(Ser11Thr), p.(Ser79Trp), p.(Trp126Arg), p.(Met258Ile), p.(Val266Met), p.(Ala273Thr), p.(Lys318Asn), p.(Thr329Met), p.(Asp358Asn), p.(Thr359Lys), and p.(Ala374Val)) alter residues that are conserved in all synapsins. The following substitutions affect amino acids that are conserved in two out of three synapsins: p.(Leu205Gln), p.(Ser212Ile), p.(Arg420Gln), and p.(Arg556Cys). The two remaining missense variants, p.(Ala310Asp) and p.(Ala550Thr), affect residues that are specific for SYN1a and SYN1b (Supplementary Figure S2). According to the SpliceAI neural network, none of the missense substitutions is predicted to affect splice site junctions (Supplementary Table S1). We subsequently employed DynaMut2 and mCSM to predict the putative effects of missense substitutions on protein stability. The predictions of these two bioinformatic tools are in agreement with each other for all the analyzed variants and suggest that four variants (p.(Trp126Arg), p.(Lys318Asn), p.(Leu205Gln), and p.(Arg114Gly)) might lead to destabilization of the SYN1 protein (Supplementary Table S1).

**FIGURE 2:**
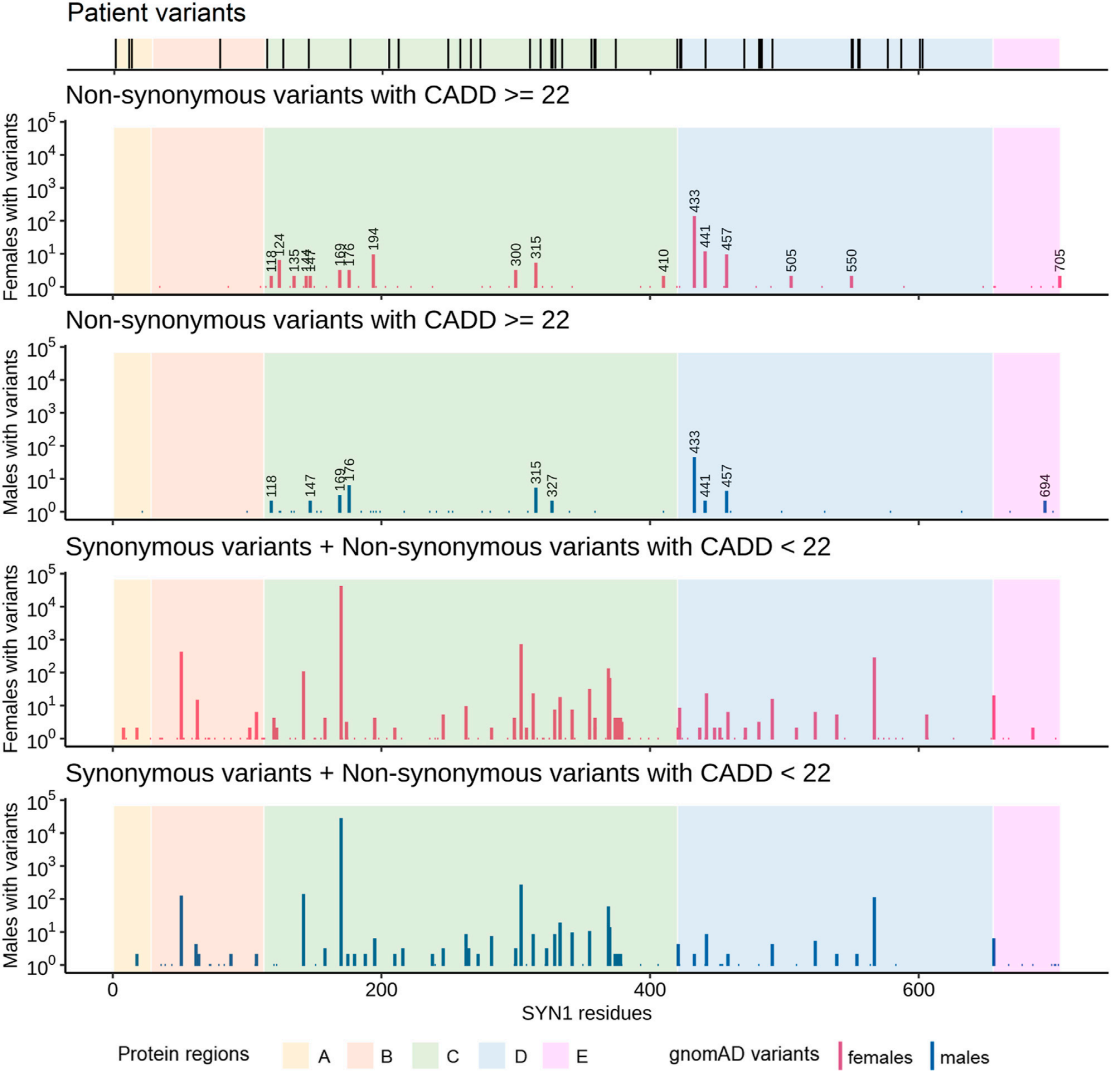
Comparison of the distribution of the *SYN1* variants identified in our cohort and/or in the literature with the variants reported in gnomAD. The number of individuals with gnomAD variants was plotted for each combined codon. gnomAD variants were stratified by both sex and damage potential (based on variant type and CADD score). Specifically, gnomAD variants were subdivided in the following two groups: one comprising synonymous variants and non-synonymous variants with CADD scores lower than 22 and one comprising non-synonymous variants with CADD scores equal or higher than 22 (putatively damaging gnomAD variants). Numbers above bars represent the positions of residues affected by putatively damaging non-synonymous variants in at least two males or two females. Males are depleted in putatively damaging *SYN1* variants compared to females (*p* = 7.3 × 10^–4^, OR = 0.67, Fisher’s test). Regions A and B are almost devoid of putatively damaging variants in gnomAD.

All putatively disease-causing *SYN1* variants are either absent from gnomAD or present at a very low allele frequency (<0.0001%). All *SYN1* variants available in gnomAD were subsequently retrieved to compare a large population of males and females and the distribution of variants across different protein regions. Putatively damaging *SYN1* variants (non-synonymous variants with CADD score ≥22) affect 78 residues, while non-deleterious variants (synonymous or non-synonymous variants with CADD score <22) alter codons corresponding to 140 residues (Figure 2). Notably, males are depleted in putatively damaging *SYN1* variants compared to females (*p* = 7.3 × 10^–4^, OR = 0.67, Fisher’s test). Regions A and B are almost devoid of putatively damaging gnomAD variants while in the other protein regions, 17 residues are altered by putatively damaging variants in multiple female individuals whereas only 10 are altered in multiple males (Figure 2).

### *SYN1* variants predominantly affect males and result in clinical heterogeneity

This cohort comprises 29 males and two females with *SYN1* variants (Supplementary Table S2). Taking previously published patients into account, the number of individuals with *SYN1*-related neurodevelopmental disorders for whom clinical information is available is 83 (71 males and 12 females; Table 1) (Garcia et al. 2004; Fassio et al. 2011; Nguyen et al. 2015; Guarnieri et al. 2017; Peron et al. 2018; Fernandez-Marmiesse et al. 2019; Darvish et al. 2020; Ibarluzea et al. 2020; Accogli et al. 2021; Mojarad et al. 2021; van der Ven et al. 2021; Xiong et al. 2021; Yang et al. 2020; Zhou et al. 2021).

**TABLE 1:** Main clinical features of individuals with *SYN1* variants.

Individual						Clinical features									
Source	Individual ID	Sex	cDNA	Protein	Origin	DD	ID	Behavioral issues	ASD	ADHD	Other behavioral issues	Epilepsy	Age of onset (years)	Reflex seizures	Trigger
This cohort	1 (Fam 1, IV-1)	M	c.2del	p.?	mat	−	+	+	−	+	+	+	8	+	rubbing with towel, defecation
This cohort	2 (Fam 1, IV-2)	M	c.2del	p.?	mat	−	+	+	−	−	+	+	NA	+	warm bath
This cohort	3 (Fam 1, III-4)	M	c.2del	p.?	mat	+	+	+	+	−	+	+	NA	−	None
This cohort	4 (Fam 2)	M	c.1258dup	p.(Arg420Profs*264)	mat	+	+	+	+	−	+	+	NA	−	None
This cohort	5 (Fam 3, IV-3)	M	c.975del	p.(Tyr326Thrfs*2)	mat	+	+	+	+	−	+	+	7	+	emotions and lightning
This cohort	6 (Fam 3, IV-5)	M	c.975del	p.(Tyr326Thrfs*2)	mat	−	NA	+	−	+	−	+	3	−	none
This cohort	7 (Fam 4)	M	c.1729del	p.(Ala577Profs*90)	mat	+	+	+	+	−	−	+	5	+	bathing, sleep, illness, digestive troubles
This cohort	8 (Fam 5, III-9)	M	c.1001del	p.(Asn334Thrfs*74)	mat	+	+	+	+	−	+	+	12	−	none
This cohort	9 (Fam 5, II-4)	M	c.1001del	p.(Asn334Thrfs*74)	mat	+	+	NA	NA	NA	NA	+	11	−	none
This cohort	10 (Fam 5, II-2)	M	c.1001del	p.(Asn334Thrfs*74)	mat	+	+	+	−	−	+	NA	NA	NA	NA
This cohort	11 (Fam 5, II-1)	M	c.1001del	p.(Asn334Thrfs*74)	mat	+	+	NA	NA	NA	NA	+	NA	NA	NA
This cohort	12 (Fam 6, III-1)	M	c.1439dup	p.(Leu481Ilefs*203)	mat	+	+	NA	NA	NA	NA	+	13	−	none
This cohort	13 (Fam 7, III-1)	M	c.1794_1906del	p.(Thr601Glufs*45)	mat	+	+	+	+	−	+	+	0.5	−	none
This cohort	14 (Fam 8, II-1)	M	c.1447C>T	p.(Gln483*)	mat	+	−	+	−	+	+	+	8	+	shower with warm water
This cohort	15 (Fam 8, II-2)	M	c.1447C>T	p.(Gln483*)	mat	−	NA	+	−	−	+	+	13	+	shower with warm water
This cohort	16 (Fam 8, II-3)	M	c.1447C>T	p.(Gln483*)	mat	−	NA	−	−	−	−	+	11	+	shower with warm water
This cohort	17 (Fam 9)	M	c.1321dup	p.(Ala441Glyfs*243)	mat	+	+	−	−	−	−	+	0.8	+	hot water
This cohort	18 (Fam 10, III-2)	M	c.1072G>A	p.(Asp358Asn)	mat	+	−	+	+	−	+	+	1	−	none
This cohort	19 (Fam 11)	F	c.986C>T	p.(Thr329Met)	de novo	+	−	+	−	−	+	−	NR	−	NR
This cohort	20 (Fam 12, III-1)	M	c.1264C>T	p.(Arg422*)	mat	+	+	NA	NA	NA	NA	+	0.6	+	showering and using the swimming pool
This cohort	21 (Fam 13)	M	c.954G>T	p.(Lys318Asn)	de novo	+	−	+	+	+	+	+	9	−	none
This cohort	22 (Fam 14)	M	c.614T>A	p.(Leu205Gln)	mat	+	−	+	−	−	+	+	11	+	defecation
This cohort	23 (Fam 15)	M	c.774G>T	p.(Met258Ile)	mat	+	−	+	−	−	+	+	NA	+	hyperpnea
This cohort	24 (Fam 16, III-2)	M	c.745C>T	p.(Gln249*)	mat	+	NA	+	+	−	−	−	NR	−	NR
This cohort	25 (Fam 17)	M	c.340A>G	p.(Arg114Gly)	mat	+	+	+	−	−	+	+	1	+	stroboscope
This cohort	26 (Fam 18)	M	c.39del	p.(Phe13Leufs*10)	mat	−	+	−	−	−	−	+	10	+	toothbrushing, warm bath or shower
This cohort	27 (Fam 19, II-1)	M	c.1121C>T	p.(Ala374Val)	mat	+	−	+	+	+	−	−	NR	−	NR
This cohort	28 (Fam 19, II-3)	M	c.1121C>T	p.(Ala374Val)	mat	+	+	+	+	−	+	−	NR	−	NR
This cohort	29 (Fam 20)	F	c.980+43_981del	p.(Met327Ilefs*81)	pat	+	+	NA	NA	NA	NA	−	NR	−	NR
This cohort	30 (Fam 21)	M	c.614_616dup	p.(Leu205dup)	de novo	+	+	−	−	−	−	+	1	+	warm bath
This cohort	31 (Fam 22, III-2)	M	c.528-2A>T	p.?	mat	−	+	+	−	+	−	+	1.5	+	contact with water
van der Ven et al. (2021)	Paed234	F	c.32G>C	p.(Ser11Thr)	de novo	NA	NA	−	−	−	−	+	0.6	NA	NA
Guarnieri et al. (2017)	IV-2	M	c.236C>G	p.(Ser79Trp)	mat	+	+	NA	NA	NA	NA	−	NR	−	NR
Guarnieri et al. (2017)	IV-11	F	c.236C>G	p.(Ser79Trp)	mat		+	NA	NA	NA	NA	−	NR	−	NR
Guarnieri et al. (2017)	V-19	M	c.236C>G	p.(Ser79Trp)	mat	+	+	+	NA	NA	+	−	NR	−	NR
Guarnieri et al. (2017)	V-22	M	c.236C>G	p.(Ser79Trp)	mat	+	+	NA	NA	NA	NA	−	NR	−	NR
Fernández-Marmiesse et al. (2019)	740	M	c.376T>A	p.(Trp126Arg)	mat	+	+	NA	NA	NA	NA	+	5	NA	NA
Accogli et al. (2021)	Fam IV, II-2	M	c.436-1G>C	p.?	mat	−	NA	NA	NA	NA	NA	+	8	+	showering (pouring water over the head)
Peron et al. (2018)	II-4	M	c.527+1G>T	p.?	mat	NA	+	NA	NA	NA	NA	+	NA	+	contact with water (particularly hot water)
Peron et al. (2018)	III-1	M	c.527+1G>T	p.?	mat	NA	+	NA	NA	NA	NA	+	8	+	contact with water (particularly hot water)
Accogli et al. (2021)	Fam III, II-1	M	c.774+2T>C	p.?	mat	+	+	+	+	−	+	+	7	+	showering, rubbing with towel
Ibarluzea et al. (2020)	Fam ID1402, III-1	M	c.796G>A	p.(Val266Met)	mat	NA	+	+	+	−	−	NA	NA	NA	NA
Ibarluzea et al. (2020)	Fam ID1402, II-2	M	c.796G>A	p.(Val266Met)	mat	NA	−	+	−	−	+	NA	NA	NA	NA
Accogli et al. (2021)	Fam 7, II-3	M	c.929C>A	p.(Ala310Asp)	mat	−	−	NA	NA	NA	NA	+	1	+	during or after bathing, hair washing
Garcia et al. (2004)	II-5	M	c.1067G>A	p.(Trp356*)	mat	−	−	NA	NA	NA	NA	+	1	NA	NA
Garcia et al. (2004)	III-2	M	c.1067G>A	p.(Trp356*)	mat	−	−	NA	NA	NA	NA	+	7	NA	NA
Garcia et al. (2004)	III-3	M	c.1067G>A	p.(Trp356*)	mat	−	+	+	NA	NA	+	+	11	NA	NA
Garcia et al. (2004)	III-7	M	c.1067G>A	p.(Trp356*)	mat	−	−	NA	NA	NA	NA	+	18	NA	NA
Garcia et al. (2004)	III-10	M	c.1067G>A	p.(Trp356*)	mat		+	+	NA	NA	+	−	NR	−	NR
Garcia et al. (2004)	IV-1	M	c.1067G>A	p.(Trp356*)	mat	−	−	+	NA	NA	+	+	16	+	while falling asleep or during sleep
Garcia et al. (2004)	IV-4	M	c.1067G>A	p.(Trp356*)	mat	−	−	NA	NA	NA	NA	+	3	NA	NA
Garcia et al. (2004)	IV-5	M	c.1067G>A	p.(Trp356*)	mat	NA	+	+	+	−	+	−	NR	−	NR
Xiong et al. (2021)	Fam A	M	c.1076C>A	p.(Thr359Lys)	mat	+	+	NA	NA	NA	NA	+	1	+	fever
Darvish et al. (2020)	Fam ID-05; II-4	M	c.1259G>A	p.(Arg420Gln)	mat	NA	+	+	+	NA	−	−	NR	−	NR
Darvish et al. (2020)	Fam ID-05; II-5	M	c.1259G>A	p.(Arg420Gln)	mat	NA	+	+	+	NA	−	−	NR	−	NR
Accogli et al. (2021)	Fam 1, II-1	M	c.1264C>T	p.(Arg422*)	mat	NA	NA	+	NA	+	−	+	5	+	showering, rubbing with towel, watching his sister having a shower
Accogli et al. (2021)	Fam 10, IV-2	M	c.1266del	p.(Gln423Serfs*244)	NA	NA	+	+	+	NA	−	+	15	+	immersion of the feet in water and febrile events
Accogli et al. (2021)	Fam 5, II-1	M	c.1406dup	p.(Pro470Alafs*214)	mat	+	NA	+	NA	+	−	+	1.5	+	bathing
Accogli et al. (2021)	Fam 5, II-2	M	c.1406dup	p.(Pro470Alafs*214)	mat	+	NA	+	NA	+	−	+	1	+	bathing
Accogli et al. (2021)	Fam 5, II-3	F	c.1406dup	p.(Pro470Alafs*214)	mat	+	NA	+	+	NA	−	+	2	+	bathing
Accogli et al. (2021)	Fam 2, III-1	M	c.1439dup	p.(Leu481Ilefs*203)	mat	NA	NA	+	NA	NA	+	+	2	+	bathing
Accogli et al. (2021)	Fam 2, II-3	M	c.1439dup	p.(Leu481Ilefs*203)	de novo	NA	NA	NA	NA	NA	NA	+	NA	−	None
Xiong et al. (2021)	Fam B	M	c.1444C>T	p.(Gln482*)	mat	NA	+	+	NA	NA	+	+	NA	NA	NA
Accogli et al. (2021)	Fam 6, II-4	M	c.1472_1473ins	p.(Gln491Hisfs*193)	mat	+	+	+	+	+	−	+	4.5	+	bathing, showering, haircutting, fingernail clipping, watching someone while bathing, thinking of bathing
Accogli et al. (2021)	Fam 8, II-1	M	c.1647_1650dup	p.(Ser551Argfs*134)	mat	+	+	+	+	+	+	+	0.7	+	bathing, showering, fingernail clipping
Fassio et al. (2011)	26755	M	c.1648G>A	p.(Ala550Thr)	NA	NA	NA	NA	NA	NA	NA	+	NA	NA	NA
Fassio et al. (2011)	14026	F	c.1648G>A	p.(Ala550Thr)	NA	NA	NA	NA	NA	NA	NA	+	NA	NA	NA
Fassio et al. (2011)	23679	F	c.1648G>A	p.(Ala550Thr)	NA	NA	NA	+	+	NA	NA	+	NA	NA	NA
Fassio et al. (2011)	17546	M	c.1648G>A	p.(Ala550Thr)	NA	NA	NA	+	+	NA	NA	−	NR	−	NR
Mojarad et al. (2021)	55	F	c.1648G>A	p.(Ala550Thr)	NA	NA	+	+	NA	NA	+	NA	NA	NA	NA
Nguyen et al. (2015)	V-5	F	c.1663C>T	p.(Gln555*)	mat	NA	NA	NA	NA	NA	NA	+	3	+	fever
Nguyen et al. (2015)	V-4	M	c.1663C>T	p.(Gln555*)	mat	NA	+	NA	NA	NA	NA	+	1.5	+	face rubbing with wet towel or showering
Nguyen et al. (2015)	VI-1	M	c.1663C>T	p.(Gln555*)	mat	NA	+	NA	NA	NA	NA	+	4	+	bathing, showering
Nguyen et al. (2015)	VI-25	M	c.1663C>T	p.(Gln555*)	mat	NA	+	NA	NA	NA	NA	+	13	+	during shower or while testing temperature of shower
Nguyen et al. (2015)	VI-5	F	c.1663C>T	p.(Gln555*)	mat	NA	NA	NA	NA	NA	NA	+	NA	+	fever
Nguyen et al. (2015)	VI-15	M	c.1663C>T	p.(Gln555*)	mat	NA	+	NA	NA	NA	NA	+	14	+	after shower/bath
Nguyen et al. (2015)	VII-1	M	c.1663C>T	p.(Gln555*)	mat	NA	+	+	+	NA	NA	+	4	+	bathing and nail clipping
Nguyen et al. (2015)	VII-2	M	c.1663C>T	p.(Gln555*)	mat	NA	+	+	+	NA	NA	+	9	+	after shower/bath
Yang et al. (2020)	Fam 4, II-1	F	c.1666C>T	p.(Arg556Cys)	NA	+	NA	NA	NA	NA	NA	+	2	NA	NA
Accogli et al. (2021)	Fam 9, II-2	F	c.1760_1771dup	p.(Arg587_Pro590dup)	mat	+	+	+	NA	+	NA	+	5	+	bathing or showering
Zhou et al. (2021)	III-2	M	c.1807C>T	p.(Gln603*)	mat	−	NA	NA	NA	NA	NA	+	NA	+	toothbrushing
Zhou et al. (2021)	II-1	M	c.1807C>T	p.(Gln603*)	NA	NA	NA	NA	NA	NA	NA	+	26	+	toothbrushing
Zhou et al. (2021)	II-4	M	c.1807C>T	p.(Gln603*)	NA	NA	NA	+	NA	NA	+	NA	NA	NA	NA

DD, developmental delay; ID, intellectual disability; ASD, autism spectrum disorder; ADHD, attention deficit hyperactivity disorder; NA, not assessed; NR, not relevant; Fam, family; Dup, duplication; Mat, maternal; Pat, paternal.

Most *SYN1* variants were identified in families with multiple affected family members. Four *SYN1* variants arose *de novo* in the index patient (Table 1). One additional *de novo* variant in an unaffected mother was previously reported (Accogli et al. 2021). Of the four *de novo* variants, two were identified in female individuals (Individual 19 in our cohort and Paed234 in van der Ven et al. 2021) and two in males, both part of our cohort. One male subject was hemizygous for the variant (Individual 21), while the other male individual displayed somatic mosaicism in blood (Individual 30) (Supplementary Table S2). Remarkably, somatic mosaicism in blood was identified also in the unaffected father of the female Individual 29.

**FIGURE 3:**
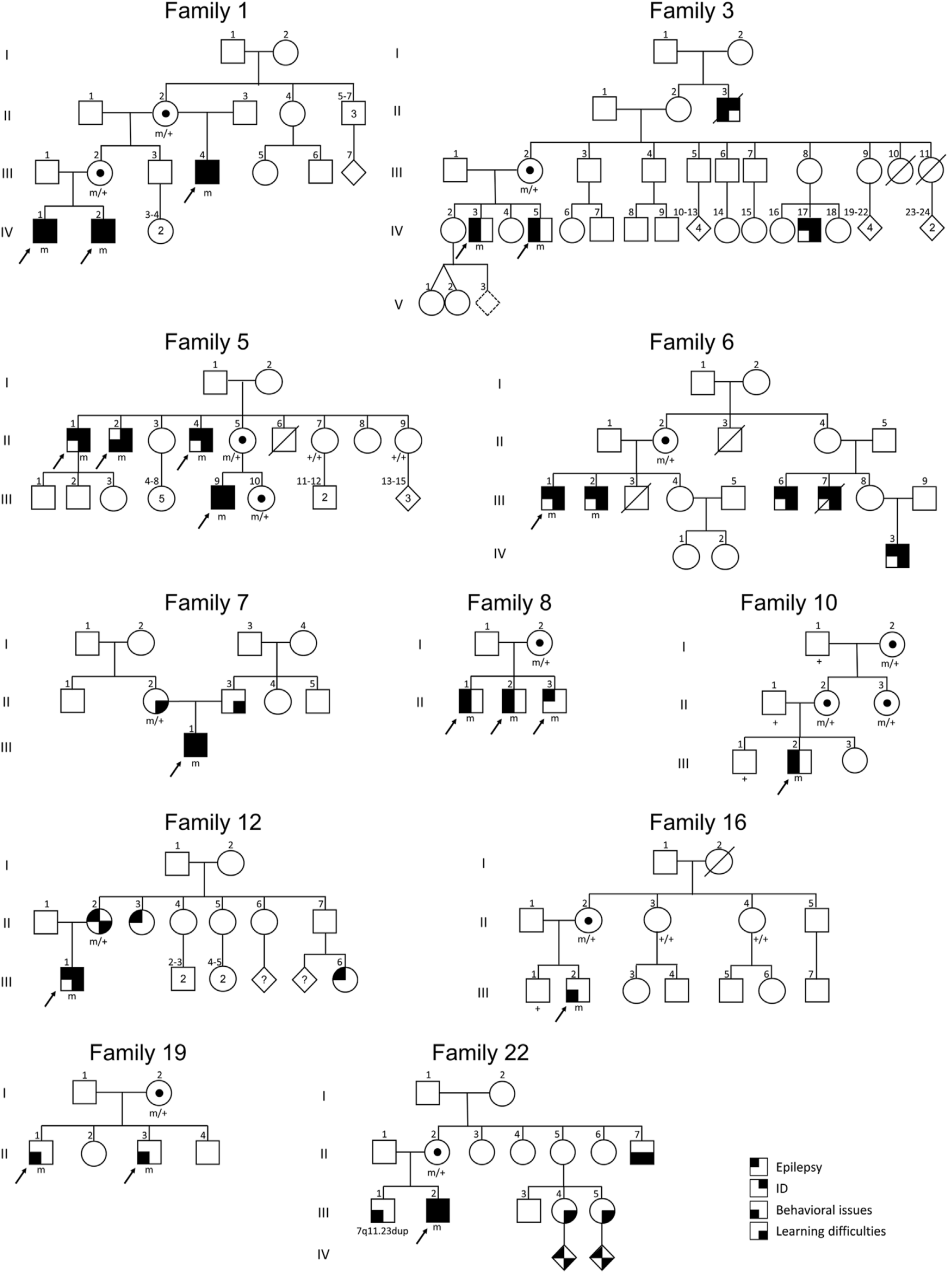
Pedigrees of the *SYN1* familial cases of our cohort. Black arrows indicate the index cases initially referred to the genetic center. Individuals within the same family tree are variably affected, pointing to the existence of incomplete penetrance and clinical heterogeneity.

Overall, this study brings the total number of families with *SYN1* variants to 50 (22 from our cohort and 28 from the literature). Pedigrees of the families from our cohort are depicted in Figure 3. In familial cases, males show a more homogeneous clinical presentation at the more severe end of the *SYN1*-phenotypic spectrum. Conversely, females can display a broad clinical spectrum, ranging from unaffected to equally affected as males. Nevertheless, male individuals within the same family can also show different phenotypes or severity of the displayed clinical features, leading to the hypothesis that additional factors might act as phenotypic modifiers.

When assessed, X-inactivation analysis of the healthy female carriers within families did not reveal any skewing (Supplementary Table S2). Notably, Individual 29, who inherited the *SYN1* variant from her mosaic father, displays complete skewing of the X-inactivation.

Altogether, our data suggest that *SYN1* variants predominantly affect male individuals, whereas females exhibit a wider clinical heterogeneity and incomplete penetrance, possibly related to the status of X-inactivation in the brain. Extensive intrafamilial variability is also reported.

### *SYN1*-related disorders are characterized by developmental delay, intellectual disability, behavioral disturbances, and epilepsy

A detailed clinical description of each individual in our cohort was provided by the referring physician and can be found in Supplementary Table S2. A summary of the main clinical features of all individuals (from our cohort and the literature) is provided in Table 1.

**FIGURE 4:**
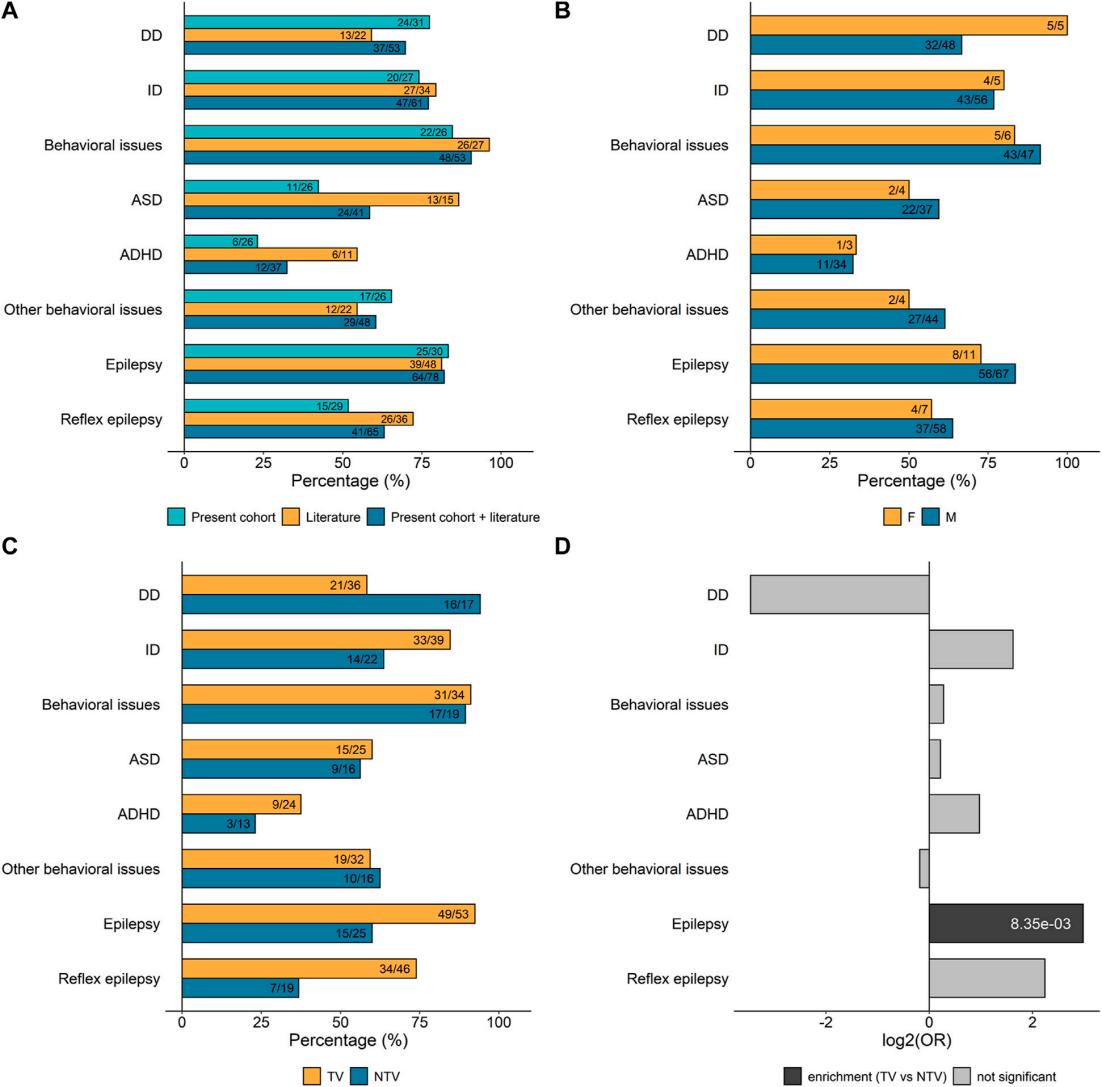
Frequency of the main clinical features associated with *SYN1* variants. Numbers inside the bars are ratios between the number of patients presenting with the specific clinical sign and the number of patients for which information on that specific feature was available. **(A)** Percentage of *SYN1* patients in our cohort and/or in the literature manifesting the clinical features of interest. **(B)** Percentage of male and female *SYN1* patients manifesting specific clinical features. Data from patients in our cohort and the literature were combined. **(C)** Percentage of patients with either truncating variants (TV) or non-truncating variants (NTV) manifesting specific clinical features. Data from patients in our cohort and the literature were combined. TV: nonsense, frameshift and splicing variants; NTV: missense substitutions and in-frame duplications. **(D)** Per clinical feature enrichment/depletion of TV *versus* NTV. Only significant *p*-values are shown inside bars (Fisher’s test followed by Bonferroni correction for multiple testing; Supplementary Table S4). DD, developmental delay; ID, intellectual disability; ASD, autism spectrum disorder; ADHD, attention deficit hyperactivity disorder.

In our cohort, pregnancy and infancy were typically uneventful. Birth parameters were similar to those of the general population. Developmental delay (DD) was reported in 77% (24/31) of the individuals (Figure 4A), typically affecting speech acquisition more than gross motor development. Walking independently was achieved at a median age of 17.5 months (range 12–36 months), whereas the median age of pronouncing the first words was 24 months (ranging from 12 months to 12 years of age). At the latest clinical examination, ten individuals (age range: 3.5–50 years) were either non-verbal or could only pronounce a few words or short sentences. ID was present in 74% (20/27) of the subjects for whom assessment was possible. The degree of ID could be assessed for 16 of the 20 individuals and was mild in ten, moderate in three, and severe in three. Six individuals showed signs of regression; in five of them, regression started after the onset of epilepsy. Epilepsy was one of the cardinal features, reported in 83% (25/30) of the individuals. Triggers of seizures were reported in 60% (15/25) of those with epilepsy. Behavioral disturbances were present in 85% (22/26) of the individuals. More specifically, ASD was diagnosed in 42% (11/26) and attention deficit hyperactivity disorder (ADHD) in 23% (6/26) of the total number of individuals of our cohort. Additional behavioral issues were observed in 65% (17/26) of the cases and comprise poor eye contact, aggressivity, impulsivity and sleep disorders. At the latest clinical examination, weight and height were mostly in the normal range for a given age and sex. Five individuals exhibited macrocephaly (>2 SD). When performed, brain magnetic resonance imaging (MRI) and neurologic examinations were normal. Specifically, MRI was normal in 70% (14/20) of the individuals of our cohort and the clinical neurological examination in 88% (22/25). Facial dysmorphic features were rarely reported and were rather unspecific.

The frequency of the main clinical features (DD, ID, behavioral disturbances, and epilepsy) was subsequently calculated on the total number of individuals with *SYN1* variants for whom clinical information was available: altogether, DD was present in 70% of the total subjects (37/53), ID in 77% (47/61), ASD in 59% (24/41), ADHD in 32% (12/37), epilepsy in 82% (64/78), and reflex epilepsy in 63% (41/65) (Figure 4A). Importantly, individuals with *SYN1* variants can present with a variable combination and severity of these clinical signs.

Next, we investigated whether the clinical features of interest are enriched in association with a specific variant type or with the sex of the individual. Males represent the vast majority of affected individuals, with 71 males and 12 females with *SYN1* putatively pathogenic variants reported so far. Although hemizygous males tend to show a more homogeneous and severe clinical presentation, no specific clinical feature was found to be enriched in males compared to females (Figure 4B, Supplementary Table S3). We then compared the frequency of ID, DD, behavioral disturbances, and epilepsy in individuals with truncating variants (TV; frameshift, nonsense, start-loss, and splicing variants) and non-truncating variants (NTV; including both missense substitutions and in-frame duplications) (Figures 4C,D). Subjects with TV presented more frequently with seizures than subjects with NTV (*p* = 8.35 × 10^–3^, Fisher Test, Supplementary Table S4). Reflex seizures were also more frequent in individuals with TV, and conversely, DD was more often observed in individuals with NTV, although these two differences were not statistically significant after adjusting for multiple testing (Figures 4C,D; Supplementary Table S4).

**FIGURE 5:**
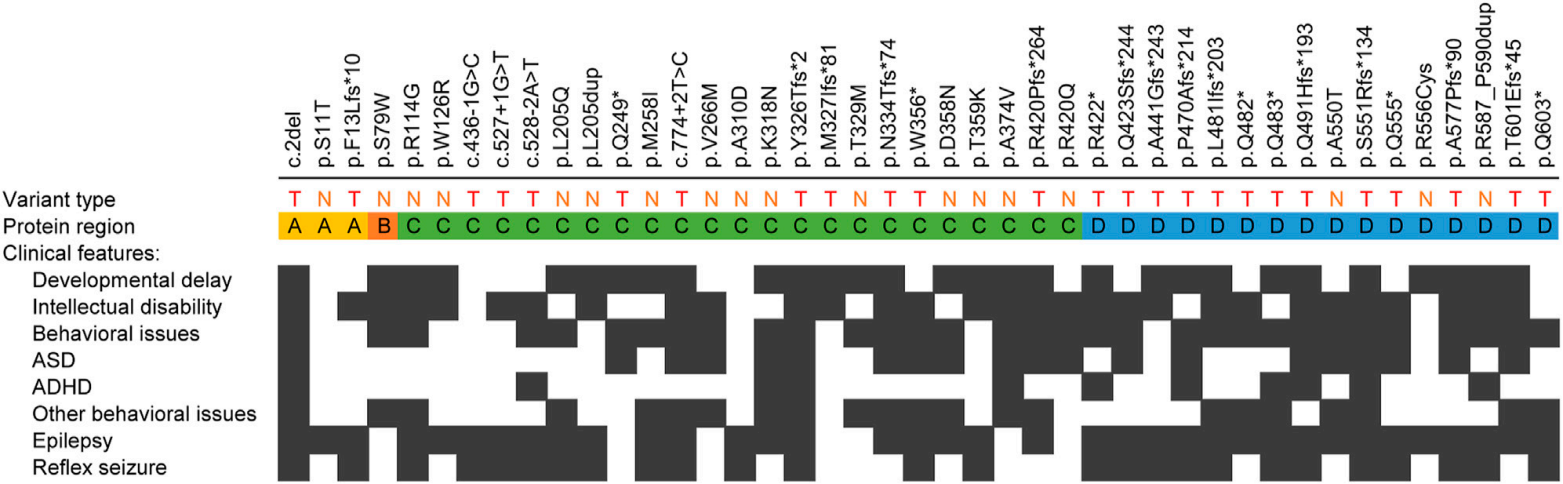
Combined clinical features observed in patients with specific *SYN1* variants. T: truncating variants (nonsense, frameshift and splicing variants); N: non-truncating variants (missense substitutions and in-frame duplications). Black squares indicate that a given clinical feature was described in association with the corresponding variant. The protein region has been subdivided in the four protein domains affected by the variants, namely domains A-D. No association can be detected between the position of the variant and the presence of a given clinical feature.

Lastly, we examined whether the presence of the main clinical features associated with *SYN1* genetic alterations correlates with the location of the variant at the protein level. As shown in Figure 5, no correlation was identified between the position of the variant or its predicted functional consequences and any specific clinical feature.

Of note, six individuals were each reported to carry a single VUS in additional genes (Supplementary Table S2). Of these, three genes (*TMTC1*, *SUPT5H*, and *MYCBP2*) had not been associated to any disease until now. The remaining three genes *KMT2C*, *RELN*, and *BRCA2*, had been previously associated with Kleefstra syndrome (OMIM #617768), Familial Temporal Lobe Epilepsy (OMIM #616436), and with different types of cancer, respectively. We cannot rule out that the presence of these subsidiary variants may have an effect on the phenotypic outcome and might therefore contribute to the *SYN1*-associated phenotypic variability.

### *SYN1* variants are frequently associated with reflex epilepsy mainly triggered by contact with water

Epilepsy represents one of the paramount features of *SYN1*-related disorders. Epileptic seizures were reported in 82% (64/78) of the total number of individuals with putatively disease-causing *SYN1* variants. The onset of seizures ranges from 6 months to 26 years of life, with a median age of onset of 7 years old. In 29% of cases with seizures, seizure-onset occurred before the second year of life and in 55% within the first 5 years of age. Ninety percent of individuals manifest their first seizures before the age of thirteen (Supplementary Figure S3). Seizure frequency is also variable, with some individuals having seizures weekly or monthly and others being seizures-free for several months. In few individuals, seizures occurred only once or represented rare events. Seizures are typically tonic-clonic (focal-to-bilateral tonic-clonic or of unknown onset) or of focal-onset with impaired awareness (Fisher et al. 2017).

Electroencephalograms of individuals with epilepsy frequently revealed focal or diffuse slow spike waves or multifocal epileptiform discharges, mainly of frontal origin. Pharmacological treatment could successfully improve epilepsy in several patients. Pharmoresistance was reported in 11 individuals of our cohort.

**FIGURE 6:**
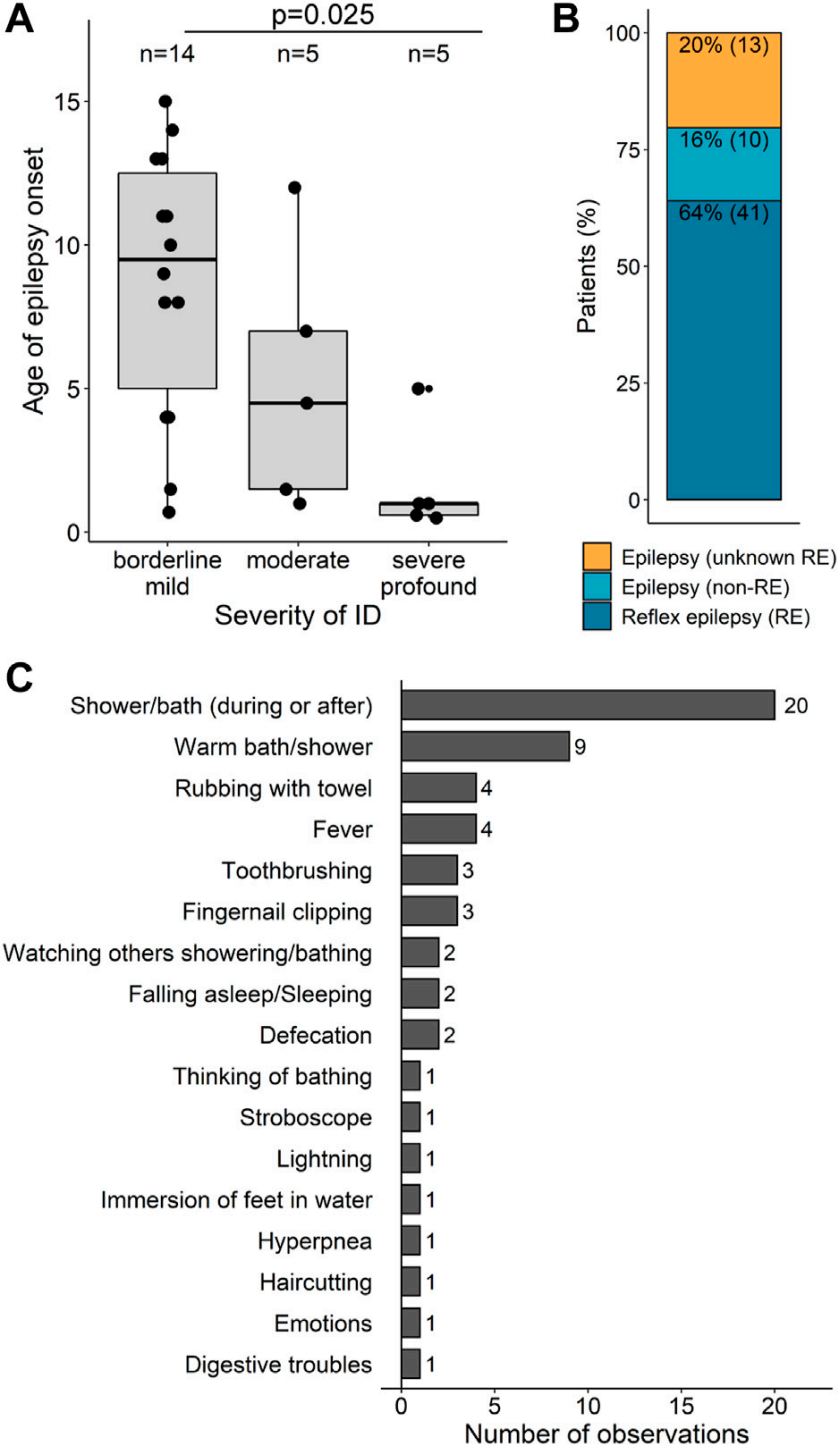
Epilepsy types and associations **(A)** Age of epilepsy onset according to the severity of ID. Patients were grouped into three categories of ID severity: borderline/mild (*n* = 14), moderate (*n* = 5) and severe/profound (*n* = 5). Each point represents one patient for which information was obtained. Box plot elements are defined as follows: center line: median; box limits: upper and lower quartiles; whiskers: 1.5× interquartile range; smaller point outside whiskers: outlier **(B)** Categorization of epilepsy types. Numbers inside the bars are shown as percentages and ratios for epilepsy patients. RE, reflex epilepsy. **(C)** Triggers reported in association with *SYN1* variants sorted from the most to the less frequent.

Neurodevelopmental comorbidities (DD, ID, and ASD) did not correlate with the occurrence of epilepsy (Supplementary Table S5). However, the age at seizure onset showed some degree of correlation with the severity of ID: in individuals with severe-to-profound ID, seizures manifested at a median age of 1 year of life (range: 5 months to 5 years), much earlier than individuals with borderline-to-mild ID (median age 9.5 years) (*p* = 0.025, Mann-Whitney Test and Bonferroni correction for multiple testing) (Supplementary Table S6, Figure 6A).

More than half of the individuals with epilepsy (41/64, 64%) had reflex seizures. Unprovoked seizures were reported in 16% of the individuals (10/64). The presence of reflex seizures could not be confirmed or excluded in the remaining 20% (13/64) (Figure 6B). The most frequent triggers reported in association with *SYN1* variants were showering, bathing, or contact with water (Figure 6C). In approximately one-third of these individuals, reflex seizures only occurred in the presence of warm water, whereas for the others the temperature of the water was irrelevant. Additional triggers include fever (n = 4), rubbing with towel (n = 4), toothbrushing (n = 3), fingernail clipping (n = 3), defecation (n = 2), falling asleep (n = 2), or even watching someone taking a shower (n = 2). Emotion, lightning, stroboscope, haircutting, thinking of bathing, immersion of feet in the water, digestive troubles, and hyperpnea were each reported as triggers in one individual each.

## Discussion

The proper formation, function, and plasticity of synapses are highly dependent on the synapsin protein family. Three related synapsin genes that can generate at least five different protein isoforms are present in mammals (Longhena et al. 2021; Sudhof et al. 1989). Synapsins regulate the distribution and availability of neurotransmitter vesicles as well as neurotransmitter release upon binding to actin, which serves as a molecular scaffold system to allow a higher concentration of synapsins in proximity of presynaptic vesicles clusters (Garcia et al. 2004; Cesca et al. 2010; Longhena et al. 2021; Sankaranarayanan, Atluri, and Ryan 2003). By this, synapsins regulate SVs trafficking between the reserve pool and the readily releasable pool (Cesca et al., 2010; Guarnieri et al., 2017). Synapsin I is also implicated in the regulation of the final steps of docking and priming of SVs to the presynaptic plasma membrane (Fassio et al. 2006). Therefore, synapsins represent one of the key players in neurotransmission.

Synapsins are enriched in the brain and poorly or non-expressed in other tissues (De Camilli et al. 1979; De Camilli, Cameron, and Greengard 1983). However, within the brain, the three synapsin genes display only partially overlapping patterns of expression (Porton, Kao, and Greengard 1999; De Camilli, Cameron, and Greengard 1983). In light of this incomplete redundancy, accurate expression and balance of synapsin isoforms are instrumental for proper cellular and brain functions.

Correspondingly, alterations of synaptic homeostasis consequent to variants in *SYN1* have been shown to lead to disease phenotypes mainly characterized by epilepsy, ID, DD, and ASD (Garcia et al. 2004; Accogli et al. 2021; Longhena et al. 2021). Due to the increasing accessibility of next-generation sequencing technologies, we were able to assemble a large cohort of individuals with *SYN1* variants through international collaborations and Genematcher, thereby expanding the clinical and molecular spectrum of *SYN1*-related disorders. DD, ID, behavioral issues, and epilepsy (particularly reflex epilepsy) were corroborated as the main features of these disorders. ASD and reflex epilepsy were found to be more frequent in previously published cohorts than in our cohort. This could be accounted for by the gene-based inclusion criteria of our cohort in comparison to the phenotype-focus of previous publications. The advantage resulting from such a genotype-first approach is the more comprehensive recognition of the phenotypic spectrum associated with variants in a given gene.

Reflex epilepsy is emblematic of *SYN1*-related disorders (Nguyen et al. 2015; Peron et al. 2018; Accogli et al. 2021; Zhou et al. 2021). Contact with water, such as during bathing or showering, is the most frequent trigger in individuals with *SYN1* variants. The temperature of the water may be relevant only for some subjects (Nguyen et al. 2015; Peron et al. 2018). As previously suggested, water temperature might play a confounding role, whereby the contact with water represents the real somatosensory stimulus that triggers the seizures (Accogli et al. 2021). Additionally, the precise contribution of water contact and temperature is complicated by other reported triggers including activities that are likely to occur around bath time. Herein, we expand on the type and number of possible triggers to include visual triggers (lighting, stroboscope lights), hyperpnea, emotions, as well as more unusual triggers such as digestive troubles or defecation. Particularly, seizures triggered by defecation were reported in two out of 15 individuals with reflex epilepsy of our cohort (13%) and warrant therefore special attention.

In our combined cohort, we did not detect enrichment of DD, ID, or ASD in individuals with epilepsy, suggesting that cognitive impairment processes are not a consequence of epileptic seizures and likely arise from parallel pathophysiological processes resulting from the *SYN1* alteration. This is in line with previous evidence showing that social impairment becomes apparent before the onset of seizures in mouse models (Greco et al. 2013). Nevertheless, an association between the age at seizure onset and the severity of ID was noted, with individuals with severe-to-profound ID having seizures at an earlier median age than subjects with less severe cognitive impairment. Additionally, loss of acquired milestones also occurred in five individuals after the onset of seizures, while only one subject showed signs of regression before seizure onset. Altogether, these data suggest that, although the distinct clinical features observed in *SYN1* individuals might develop independently from each other, seizure onset and frequency could still influence the course or the level of severity of other clinical manifestations.

We note that case ascertainment in many studies describing the clinical features of individuals with *SYN1* variants has been weighted towards individuals with epilepsy. Identification of more individuals without epilepsy and comparison of the detailed longitudinal developmental trajectories of individuals carrying pathogenic *SYN1* variants with and without epilepsy will help to clarify the role of seizures *per se* on neurodevelopment. The finding that epilepsy developed in 83% of *SYN1* variant carriers published to date suggests that early molecular diagnosis of a *SYN1* variant (for example, in children born within families known to carry a variant) should alert clinicians to the need for careful surveillance for possible seizures. Given that after a first seizure, a 10-year risk of subsequent seizures of 60% is sufficient to diagnose epilepsy and to tip the risk-benefit ratio in favor of commencing treatment (Fisher et al. 2014), the administration of anti-seizure medication after a first seizure should be considered. However, the possible ascertainment bias towards cases with epilepsy in the *SYN1* literature means that we should not yet conclude that anti-seizure medication should be commenced prior to the presentation of seizures as it is likely that a significant proportion of carriers would not benefit from it.

*SYN1* variants predominantly affect male individuals. The exact male:female ratio is however difficult to determine due to the paucity of detailed clinical information on female carriers. We have analyzed the reported phenotypes of 71 male and 12 female individuals. A higher frequency of DD and ID was observed in the limited female cohort, but this was not statistically significant. The ascertainment of the clinical features of a greater number of females with *SYN1* variants will help to clarify whether these variants present differently in male and female individuals. Overall, female carriers have been reported to be either unaffected or to display a variable number and severity of clinical signs, hence pointing to the existence of clinical heterogeneity and incomplete penetrance. When assessed, X-inactivation analyses performed on blood DNA of female carriers did not show any tendency towards preferential skewing, although this might not reflect the status in the brain, i.e. the primarily affected organ of the *SYN1*-related disorders. In our cohort, Individual 29 presented with skewed X-inactivation in blood. Whether different degrees of X-inactivation in brain tissues might account for the distinct phenotypical manifestations of the female individuals is an intriguing possibility. Notably, *SYN1* is not reported to escape X-inactivation in females (Balaton, Cotton, and Brown 2015).

Mosaicism had not been reported previously in association with *SYN1*. In our cohort, we identified two *SYN1* variants in a mosaic state in blood DNA in two male individuals. Strikingly, the mosaic variant resulted in a classic phenotype characterized by the presence of epilepsy in Individual 30, whereas no clinical features were reported for the mosaic father of Individual 29. A different percentage of the mutant allele in brain tissues might explain the different phenotypes, similarly to what has been postulated regarding X-inactivation in female carriers, and as previously described in other epilepsy disorders like *SCN1A*-related Dravet syndrome (Depienne et al. 2010).

Multiple types of alterations were identified in our cohort and the literature, including frameshift duplications or deletions, nonsense variants, splicing variants, in-frame duplications, and missense substitutions (Garcia et al, 2004; Fassio et al., 2011; Butler et al., 2017; Nguyen et al., 2015; Guarnieri et al., 2017; Rossi et al., 2017; Lindy et al., 2018; Peron et al., 2018; Fernández-Marmiesse et al., 2019; Darvish et al., 2020; Ibarluzea et al., 2020; Accogli et al., 2021; Mojarad et al., 2021; Stranneheim et al., 2021; Van der Ven et al., 2021; Xiong et al., 2021; Yang et al., 2020; Zhou et al., 2021). We also report for the first time a deletion of a single nucleotide within the first codon of the SYN1 protein (Individuals 1–3). The loss of the start codon consequent to this deletion likely leads to alternative translation initiation and subsequent generation of an N-terminally truncated protein. SYN1 N-terminus is highly conserved and depleted from putatively pathogenic alterations within the gnomAD database. Additionally, several residues of this region are pivotal for the phosphorylation-dependent regulation of SYN1 activity (Cesca et al. 2010; Longhena et al. 2021). Hence, the start-loss variant identified in our cohort might impair the ability of SYN1 to interact with its binding partners, which is likely to affect the protein’s ability to carry out its normal function in SV dynamics.

Epilepsy was more frequently observed in individuals with TV (frameshift, nonsense, and splicing variants). Accordingly, functional studies have shown that *SYN1* TV lead to higher network excitability and firing/bursting activity (Lignani et al. 2013). Decreased stability or expression of the mutated protein as well as alterations of its subcellular distribution or the inter- or intramolecular interactions were found to result in altered trafficking or release of the SVs, which could ultimately affect neural network excitability and induce the consequent epileptic phenotypes (Lignani et al. 2013; Fassio et al. 2011; Giannandrea et al. 2013).

Some missense substitutions were similarly shown to lead to reduced protein expression and altered SVs mobility or clustering (Fassio et al. 2011; Guarnieri et al. 2017; Darvish et al. 2020). Missense variants mainly fall within the category of VUS. Analysis of the conservation of the affected residues across species or synapsin isoforms together with bioinformatic predictions support a putative pathogenic role for all but one reported missense substitution. Namely, the p.(Arg114Gly) variant identified in Individual 25 of our cohort affects a residue that is poorly evolutionarily conserved, nor conserved across different synapsin proteins. For this reason, the functional/physiological relevance of this variant in the context of the individual’s phenotype remains uncertain. However, the prediction of the impact of this variant on protein stability suggests that the p.(Arg114Gly) substitution might be destabilizing. Notably, no additional disease-relevant variant was identified for this subject by targeted sequencing of an ID panel comprising 451 genes.

In conclusion, our study expands on the molecular spectrum of *SYN1* variants and improves the clinical characterization of *SYN1*-related neurodevelopmental disorders. The newly reported data and unified analysis of the clinical and molecular features of all published carriers will refine genetic counselling and the personalization of medical care for individuals with *SYN1*-related neurodevelopmental disorders. Future studies will be required to understand the mechanisms by which specific pathogenic *SYN1* variants result in a range of epileptic and neurodevelopmental phenotypes, and to clarify the mechanisms underlying the reflex seizures. These should be complemented by studies to assess the clinical and developmental trajectories of individuals with *SYN1* variants and to examine how these are affected by co-morbid seizures and medication exposure with the ultimate aim of providing precision medicine.

## Data Availability

The datasets presented in this study can be found in online repositories. The link to the repository/repositories and accession number(s) can be found below: https://www.ncbi.nlm.nih.gov/, SUB11906611 (SCV002558865 - SCV002558886).

## References

[B1] Abouelhoda M. Faquih T. El-Kalioby M. Alkuraya F. S. (2016). Revisiting the morbid genome of Mendelian disorders. Genome Biol. 17, 235. 10.1186/s13059-016-1102-1 27884173 PMC5123336

[B2] Accogli A. Wiegand G. Scala M. Cerminara C. Iacomino M. Riva A. (2021). Clinical and Genetic Features in Patients With Reflex Bathing Epilepsy. Neurology 97, e577–e586. 10.1212/WNL.0000000000012298 34078716 PMC8424500

[B3] Balaton B. P. Cotton A. M. Brown C. J. (2015). 'Derivation of consensus inactivation status for X-linked genes from genome-wide studies. Biol. Sex Differ. 6, 35. 10.1186/s13293-015-0053-7 26719789 PMC4696107

[B4] Butler K. M. da Silva C. Alexander J. J. Hegde M. Escayg A. (2017). Diagnostic Yield From 339 Epilepsy Patients Screened on a Clinical Gene Panel. Pediatr. Neurol. 77, 61–66. 10.1016/j.pediatrneurol.2017.09.003 29056246 PMC6885003

[B5] Cabana J. F. Gilbert G. Letourneau-Guillon L. Safi D. Rouleau I. Cossette P. Nguyen D. K. (2018). 'Effects of SYN1Q555X mutation on cortical gray matter microstructure. Hum. Brain Mapp. 39, 3428–48. 10.1002/hbm.24186 29671924 PMC6866302

[B6] Cambiaghi M. Cursi M. Monzani E. Benfenati F. Comi G. Minicucci F. Valtorta F. Leocani L. (2013). 'Temporal evolution of neurophysiological and behavioral features of synapsin I/II/III triple knock-out mice. Epilepsy Res. 103, 153–60. 10.1016/j.eplepsyres.2012.07.012 22846639 PMC3574234

[B7] Cavalleri G. L. Weale M. E. Shianna K. V. Singh R. Lynch J. M. Grinton B. (2007). 'Multicentre search for genetic susceptibility loci in sporadic epilepsy syndrome and seizure types: a case-control study. Lancet. Neurol. 6, 970–80. 10.1016/S1474-4422(07)70247-8 17913586

[B8] Cesca F. Baldelli P. Valtorta F. Benfenati F. (2010). 'The synapsins: key actors of synapse function and plasticity. Prog. Neurobiol. 91, 313–48. 10.1016/j.pneurobio.2010.04.006 20438797

[B9] Corradi A. Fadda M. Piton A. Patry L. Marte A. Rossi P. (2014). 'SYN2 is an autism predisposing gene: loss-of-function mutations alter synaptic vesicle cycling and axon outgrowth. Hum. Mol. Genet. 23, 90–103. 10.1093/hmg/ddt401 23956174 PMC3857945

[B10] Corradi A. Zanardi A. Giacomini C. Onofri F. Valtorta F. Zoli M. Benfenati F. (2008). Synapsin-I- and synapsin-II-null mice display an increased age-dependent cognitive impairment. J. Cell Sci. 121, 3042–51. 10.1242/jcs.035063 18713831

[B11] Darvish H. Azcona L. J. Tafakhori A. Mesias R. Ahmadifard A. Sanchez E. (2020). 'Phenotypic and genotypic characterization of families with complex intellectual disability identified pathogenic genetic variations in known and novel disease genes. Sci. Rep. 10, 968. 10.1038/s41598-020-57929-4 31969655 PMC6976666

[B12] De Camilli P. Cameron R. Greengard P. (1983). 'Synapsin I (protein I), a nerve terminal-specific phosphoprotein. I. Its general distribution in synapses of the central and peripheral nervous system demonstrated by immunofluorescence in frozen and plastic sections. J. Cell Biol. 96, 1337–54. 10.1083/jcb.96.5.1337 6404910 PMC2112636

[B13] De Camilli P. Ueda T. Bloom F. E. Battenberg E. Greengard P. (1979). 'Widespread distribution of protein I in the central and peripheral nervous systems'. Proc Natl Acad Sci U S A 76, 5977–81. 10.1073/pnas.76.11.5977 392511 PMC411776

[B14] den Dunnen J. T Dalgleish D. R MaglottMcGowan-Jordan R. K. Hart, M. S. Greenblatt, J.A. F. SmithAntonarakis T.S. E. (2016). .'HGVS Recommendations for the Description of Sequence Variants: 2016 Update. Hum Mutat 37, 564–9. 10.1002/humu.22981Roux 26931183

[B15] Depienne C. Trouillard O. Gourfinkel-An I. Saint-Martin C. Bouteiller D. Graber D. (2010). 'Mechanisms for variable expressivity of inherited SCN1A mutations causing Dravet syndrome. J. Med. Genet. 47, 404–10. 10.1136/jmg.2009.074328 20522430

[B16] Eldomery M. K. Coban-Akdemir Z. Harel T. Rosenfeld J. A. Gambin T. Stray-Pedersen A. (2017). 'Lessons learned from additional research analyses of unsolved clinical exome cases. Genome Med. 9, 26. 10.1186/s13073-017-0412-6 28327206 PMC5361813

[B17] Etholm L. Bahonjic E. Walaas S. I. Kao H. T. Heggelund P. (2012). 'Neuroethologically delineated differences in the seizure behavior of synapsin 1 and synapsin 2 knock-out mice. Epilepsy Res. 99, 252–9. 10.1016/j.eplepsyres.2011.12.004 22236379

[B18] Etholm L. Heggelund P. (2009). 'Seizure elements and seizure element transitions during tonic-clonic seizure activity in the synapsin I/II double knockout mouse: a neuroethological description. Epilepsy Behav. 14, 582–90. 10.1016/j.yebeh.2009.02.021 19236947

[B19] Fassio A. Merlo D. Mapelli J. Menegon A. Corradi A. Mete M. (2006). The synapsin domain E accelerates the exoendocytotic cycle of synaptic vesicles in cerebellar Purkinje cells. J. Cell Sci. 119, 4257–68. 10.1242/jcs.03194 17038543

[B20] Fassio A. Patry L. Congia S. Onofri F. Piton A. Gauthier J. (2011). 'SYN1 loss-of-function mutations in autism and partial epilepsy cause impaired synaptic function. Hum. Mol. Genet. 20, 2297–307. 10.1093/hmg/ddr122 21441247

[B21] Feng J. Chi P. Blanpied T. A. Xu Y. Magarinos A. M. Ferreira A. (2002). Regulation of neurotransmitter release by synapsin III. J. Neurosci. 22, 4372–80. 20026433 12040043 10.1523/JNEUROSCI.22-11-04372.2002 PMC6758821

[B22] Fernandez-Marmiesse A. Roca I. Diaz-Flores F. Cantarin V. Perez-Poyato M. S. Fontalba A. (2019). 'Rare Variants in 48 Genes Account for 42% of Cases of Epilepsy With or Without Neurodevelopmental Delay in 246 Pediatric Patients. Front. Neurosci. 13, 1135. 10.3389/fnins.2019.01135 31780880 PMC6856296

[B23] Fisher R. S. Acevedo C. Arzimanoglou A. Bogacz A. Cross J. H. Elger C. E. (2014). 'ILAE official report: a practical clinical definition of epilepsy. Epilepsia 55, 475–82. 10.1111/epi.12550 24730690

[B24] Fisher R. S. Cross J. H. French J. A. Higurashi N. Hirsch E. Jansen F. E. (2017). 'Operational classification of seizure types by the International League Against Epilepsy: Position Paper of the ILAE Commission for Classification and Terminology. Epilepsia 58, 522–30. 10.1111/epi.13670 28276060

[B25] Garcia C. C. Blair H. J. Seager M. Coulthard A. Tennant S. Buddles M. (2004). 'Identification of a mutation in synapsin I, a synaptic vesicle protein, in a family with epilepsy. J. Med. Genet. 41, 183–6. 10.1136/jmg.2003.013680 14985377 PMC1735688

[B26] Giannandrea M. Guarnieri F. C. Gehring N. H. Monzani E. Benfenati F. Kulozik A. E. Valtorta F. (2013). Nonsense-mediated mRNA decay and loss-of-function of the protein underlie the X-linked epilepsy associated with the W356× mutation in synapsin I. PLoS One 8, e67724. 10.1371/journal.pone.0067724 23818987 PMC3688603

[B27] Gitler D. Takagishi Y. Feng J. Ren Y. Rodriguiz R. M. Wetsel W. C. (2004). 'Different presynaptic roles of synapsins at excitatory and inhibitory synapses. J. Neurosci. 24, 11368–80. 10.1523/JNEUROSCI.3795-04.2004 15601943 PMC6730366

[B28] Goujon M. McWilliam H. Li W. Valentin F. Squizzato S. Paern J. Lopez R. (2010). 'A new bioinformatics analysis tools framework at EMBL-EBI. Nucleic Acids Res. 38, W695–9. 10.1093/nar/gkq313 20439314 PMC2896090

[B29] Greco B. Manago F. Tucci V. Kao H. T. Valtorta F. Benfenati F. (2013). 'Autism-related behavioral abnormalities in synapsin knockout mice. Behav. Brain Res. 251, 65–74. 10.1016/j.bbr.2012.12.015 23280234 PMC3730181

[B30] Guarnieri F. C. Pozzi D. Raimondi A. Fesce R. Valente M. M. Delvecchio V. S. (2017). 'A novel SYN1 missense mutation in non-syndromic X-linked intellectual disability affects synaptic vesicle life cycle, clustering and mobility. Hum. Mol. Genet. 26, 4699–714. 10.1093/hmg/ddx352 28973667

[B31] Hosaka M. Sudhof T. C. (1998). 'Synapsins I and II are ATP-binding proteins with differential Ca2+ regulation. J. Biol. Chem. 273, 1425–9. 10.1074/jbc.273.3.1425 9430678

[B32] Ibarluzea N. Hoz A. B. Villate O. Llano I. Ocio I. Marti I. (2020). Targeted Next-Generation Sequencing in Patients with Suggestive X-Linked Intellectual Disability. Genes (Basel), 11. 10.3390/genes11010051 PMC7017351 31906484

[B33] International League Against Epilepsy Consortium on Complex, Epilepsies (2018). 'Genome-wide mega-analysis identifies 16 loci and highlights diverse biological mechanisms in the common epilepsies. Nat. Commun. 9, 5269. 10.1038/s41467-018-07524-z 30531953 PMC6288131

[B34] Jaganathan K. Kyriazopoulou Panagiotopoulou S. McRae J. F. Darbandi S. F. Knowles D. Li Y. I. (2019). Predicting Splicing from Primary Sequence with Deep Learning. Cell 176, 535–548. 10.1016/j.cell.2018.12.015 30661751

[B35] John A. Ng-Cordell E. Hanna N. Brkic D. Baker K. (2021). 'The neurodevelopmental spectrum of synaptic vesicle cycling disorders. J. Neurochem. 157, 208–28. 10.1111/jnc.15135 32738165

[B36] Karczewski K. J. Francioli L. C. Tiao G. Cummings B. B. Alfoldi J. Wang Q. (2020). The mutational constraint spectrum quantified from variation in 141, 456 humans. Nature 581, 434–43. 10.1038/s41586-020-2308-7 32461654 PMC7334197

[B37] Lignani G. Raimondi A. Ferrea E. Rocchi A. Paonessa F. Cesca F. (2013). 'Epileptogenic Q555X SYN1 mutant triggers imbalances in release dynamics and short-term plasticity. Hum. Mol. Genet. 22, 2186–99. 10.1093/hmg/ddt071 23406870 PMC3652419

[B38] Lindy A. S. Stosser M. B. Butler E. Downtain-Pickersgill C. Shanmugham A. Retterer K. (2018). 'Diagnostic outcomes for genetic testing of 70 genes in 8565 patients with epilepsy and neurodevelopmental disorders. Epilepsia 59, 1062–71. 10.1111/epi.14074 29655203

[B39] Longhena F. Faustini G. Brembati V. Pizzi M. Benfenati F. Bellucci A. (2021). 'An updated reappraisal of synapsins: structure, function and role in neurological and psychiatric disorders. Neurosci. Biobehav. Rev. 130, 33–60. 10.1016/j.neubiorev.2021.08.011 34407457

[B40] Michetti C. Caruso A. Pagani M. Sabbioni M. Medrihan L. David G. (2017). The Knockout of Synapsin II in Mice Impairs Social Behavior and Functional Connectivity Generating an ASD-like Phenotype. Cereb. Cortex 27, 5014–23. 10.1093/cercor/bhx207 28922833

[B41] Mojarad B. A. Yin Y. Manshaei R. Backstrom I. Costain G. Heung T. (2021). Genome sequencing broadens the range of contributing variants with clinical implications in schizophrenia. Transl. Psychiatry 11, 84. 10.1038/s41398-021-01211-2 33526774 PMC7851385

[B42] Nguyen D. K. Rouleau I. Senechal G. Ansaldo A. I. Gravel M. Benfenati F. Cossette P. (2015). 'X-linked focal epilepsy with reflex bathing seizures: Characterization of a distinct epileptic syndrome. Epilepsia 56, 1098–108. 10.1111/epi.13042 26096837

[B43] Peron A. Baratang N. V. Canevini M. P. Campeau P. M. Vignoli A. (2018). 'Hot water epilepsy and SYN1 variants. Epilepsia 59, 2162–63. 10.1111/epi.14572 30390306

[B44] Pires D. E. Ascher D. B. Blundell T. L. (2014). 'mCSM: predicting the effects of mutations in proteins using graph-based signatures. Bioinformatics 30, 335–42. 10.1093/bioinformatics/btt691 24281696 PMC3904523

[B45] Porton B. Kao H. T. Greengard P. (1999). 'Characterization of transcripts from the synapsin III gene locus. J. Neurochem. 73, 2266–71. 10.1046/j.1471-4159.1999.0732266.x 10582583

[B46] Rentzsch P. Witten D. Cooper G. M. Shendure J. Kircher M. (2019). 'CADD: predicting the deleteriousness of variants throughout the human genome. Nucleic Acids Res. 47, D886-D894–D94. 10.1093/nar/gky1016 30371827 PMC6323892

[B47] Richards S. Aziz N. Bale S. Bick D. Das S. Gastier-Foster J. (2015). 'Standards and guidelines for the interpretation of sequence variants: a joint consensus recommendation of the American College of Medical Genetics and Genomics and the Association for Molecular Pathology. Genet. Med. 17, 405–24. 10.1038/gim.2015.30 25741868 PMC4544753

[B48] Rodrigues C. H. M. Pires D. E. V. Ascher D. B. (2021). 'DynaMut2: Assessing changes in stability and flexibility upon single and multiple point missense mutations. Protein Sci. 30, 60–69. 10.1002/pro.3942 32881105 PMC7737773

[B49] Rosahl T. W. Spillane D. Missler M. Herz J. Selig D. K. Wolff J. R. (1995). 'Essential functions of synapsins I and II in synaptic vesicle regulation. Nature 375, 488–93. 10.1038/375488a0 7777057

[B50] Rossi M. El-Khechen D. Black M. H. Farwell Hagman K. D. Tang S. Powis Z. (2017). 'Outcomes of Diagnostic Exome Sequencing in Patients With Diagnosed or Suspected Autism Spectrum Disorders. Pediatr. Neurol. 70, 34–43. 10.1016/j.pediatrneurol.2017.01.033 28330790

[B51] Sankaranarayanan S. Atluri P. P. Ryan T. A. (2003). 'Actin has a molecular scaffolding, not propulsive, role in presynaptic function. Nat. Neurosci. 6, 127–35. 10.1038/nn1002 12536209

[B52] Sievers F. Wilm A. Dineen D. Gibson T. J. Karplus K. Li W. (2011). 'Fast, scalable generation of high-quality protein multiple sequence alignments using Clustal Omega. Mol. Syst. Biol. 7, 539. 10.1038/msb.2011.75 21988835 PMC3261699

[B53] Sobreira N. Schiettecatte F. Valle D. Hamosh A. (2015). 'GeneMatcher: a matching tool for connecting investigators with an interest in the same gene. Hum. Mutat. 36, 928–30. 10.1002/humu.22844 26220891 PMC4833888

[B54] Stranneheim H. Lagerstedt-Robinson K. Magnusson M. Kvarnung M. Nilsson D. Lesko N. (2021). 'Integration of whole genome sequencing into a healthcare setting: high diagnostic rates across multiple clinical entities in 3219 rare disease patients. Genome Med. 13, 40. 10.1186/s13073-021-00855-5 33726816 PMC7968334

[B55] Sudhof T. C. Czernik A. J. Kao H. T. Takei K. Johnston P. A. Horiuchi A. (1989). 'Synapsins: mosaics of shared and individual domains in a family of synaptic vesicle phosphoproteins. Science 245, 1474–80. 10.1126/science.2506642 2506642

[B56] Thauvin-Robinet C. Thevenon J. Nambot S. Delanne J. Kuentz P. Bruel A. (2019). 'Secondary actionable findings identified by exome sequencing: expected impact on the organisation of care from the study of 700 consecutive tests. Eur. J. Hum. Genet. 27, 1197–214. 10.1038/s41431-019-0384-7 31019283 PMC6777608

[B63] The UniProt Consortium (2021). UniProt: the universal protein knowledgebase in 2021. Nucleic Acids Res 49, D480–D89. 33237286 10.1093/nar/gkaa1100 PMC7778908

[B57] Turner T. N. Wilfert A. B. Bakken T. E. Bernier R. A. Pepper M. R. Zhang Z. (2019). 'Sex-Based Analysis of De Novo Variants in Neurodevelopmental Disorders. Am. J. Hum. Genet. 105, 1274–85. 10.1016/j.ajhg.2019.11.003 31785789 PMC6904808

[B58] Ueda T. Greengard P. (1977). 'Adenosine 3':5'-monophosphate-regulated phosphoprotein system of neuronal membranes. I. Solubilization, purification, and some properties of an endogenous phosphoprotein. J. Biol. Chem. 252, 5155–63. 10.1016/s0021-9258(17)40170-0 194903

[B59] van der Ven A. T. Johannsen J. Kortum F. Wagner M. Tsiakas K. Bierhals T. (2021). 'Prevalence and clinical prediction of mitochondrial disorders in a large neuropediatric cohort. Clin. Genet. 100, 766–70. 10.1111/cge.14061 34490615

[B60] Xiong J. Duan H. Chen S. Kessi M. He F. Deng X. (2021). 'Familial SYN1 variants related neurodevelopmental disorders in Asian pediatric patients. BMC Med. Genomics 14, 182. 10.1186/s12920-021-01028-4 34243774 PMC8272254

[B61] Yang J. O. Choi M. H. Yoon J. Y. Lee J. J. Nam S. O. Jun S. Y. (2020). Corrigendum: Characteristics of Genetic Variations Associated With Lennox-Gastaut Syndrome in Korean Families. Front. Genet. 11, 669107. 10.3389/fgene.2021.669107 PMC7874053 33584793

[B62] Zhou Q. Wang J. Xia L. Li R. Zhang Q. Pan S. (2021). 'SYN1 Mutation Causes X-Linked Toothbrushing Epilepsy in a Chinese Family. Front. Neurol. 12, 736977. 10.3389/fneur.2021.736977 34616357 PMC8488375

