# Supplementary material for: The different clinical facets of SYN1-related neurodevelopmental disorders
Source: Front Cell Dev Biol. 2022 Dec 8;10:1019715. doi: 10.3389/fcell.2022.1019715 (PMC9773998; doi:10.3389/fcell.2022.1019715)
Supplement: Supplementary file 1 [file DataSheet1.PDF]

Supplementary Material

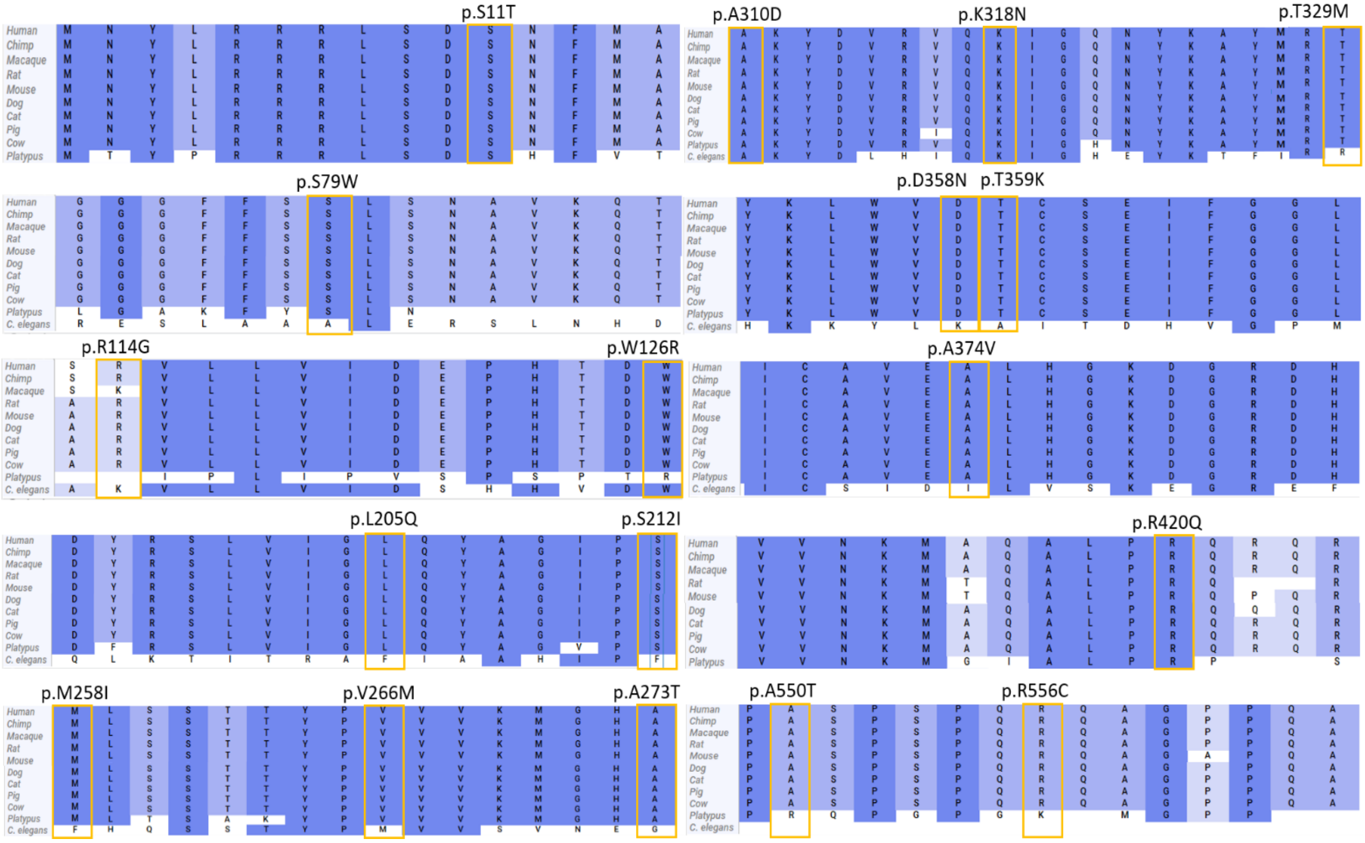

Supplementary Figure 1. Conservation of the affected amino acids across different species. Alignment of the SYN1 protein sequence in 11 species (human, chimp, macaque, rat, mouse, dog, cat, pig, cow, platypus, and C. elegans) was performed with the Alamut Visual Plus^{TM} software. This analysis shows that almost all residues affected by likely pathogenic or VUS missense substitutions are evolutionary conserved. p.Arg114 represents the only exception.

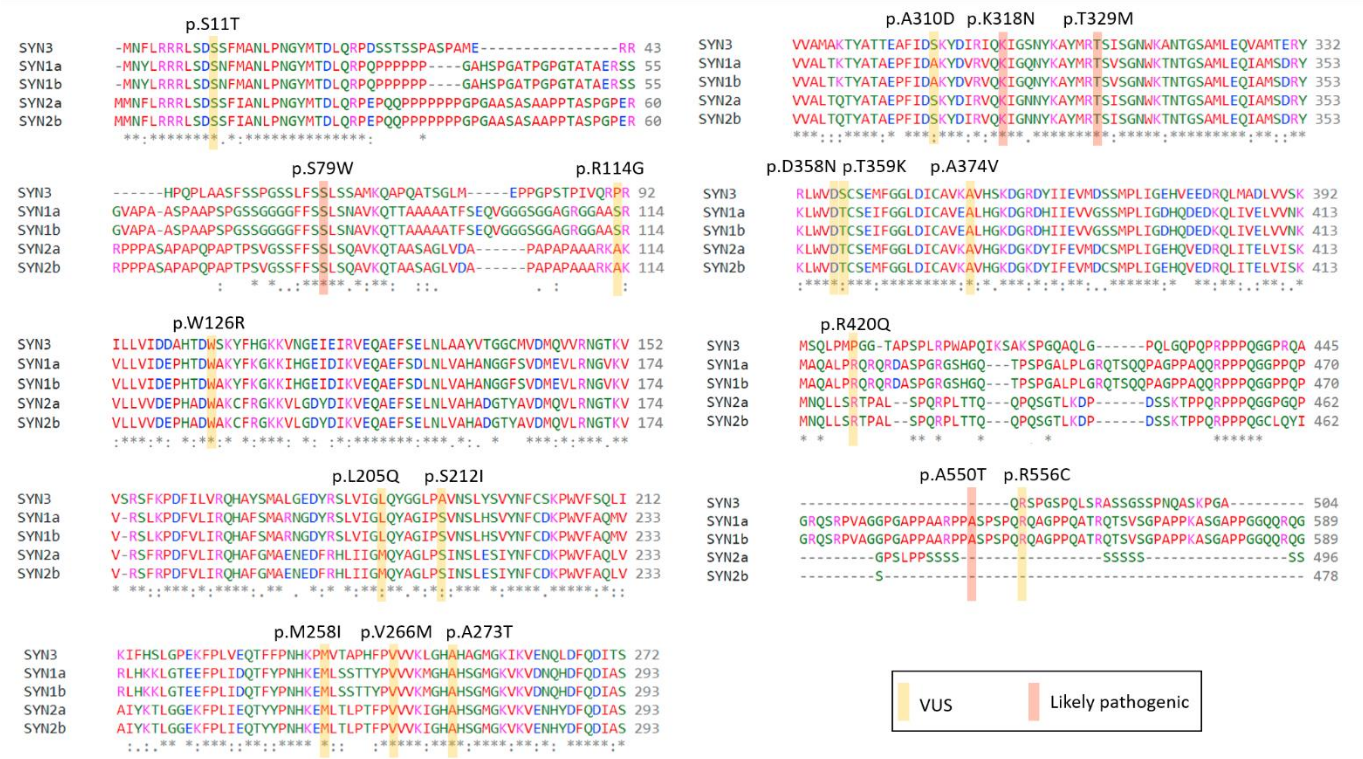

Supplementary Figure 2. Conservation of the affected amino acids across synapsin isoforms. Alignment of the protein sequence of all synapsin isoforms (SYN1a, SYN1b, SYN2a, SYN2b, SYN3) was performed with ClustalOmega. VUS missense substitutions are highlighted in yellow and likely pathogenic variants in red. Eleven missense variants (p.S11T, p.S79W, p.W126R, p.M258I, p.V266M, p.A273T, p.K318N, p.T329M, p.D358N, p.T359K, and p.A374V) alter residues that are conserved across all synapsin isoforms. The following substitutions affect amino acids that are common to two out of three synapsins: p.L205Q (SYN1 and SYN3), p.S212I (SYN1 and SYN2), p.R420Q (SYN1 and SYN2), and p.R556C (SYN1 and SYN3). The two remaining missense variants, p.A310D and p.A550T, affect residues that are specific to SYN1a and SYN1b.

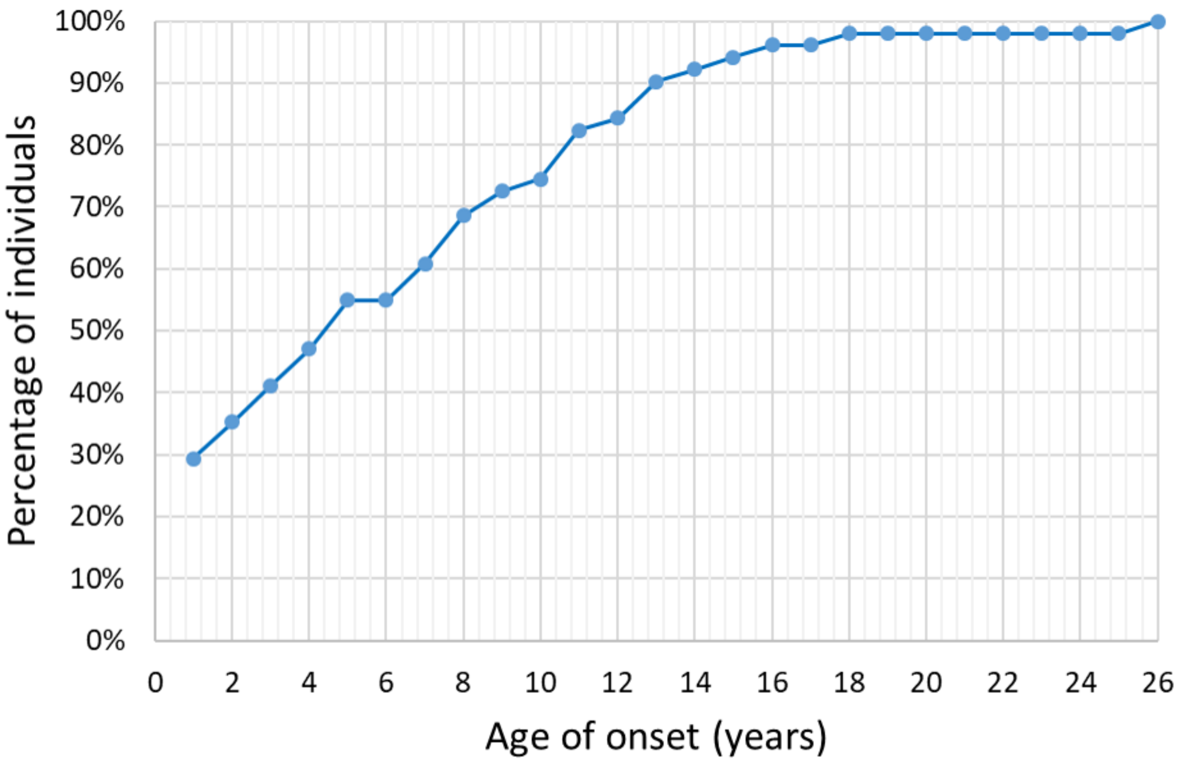

Supplementary Figure 3. Trajectory of epilepsy onset in individuals with *SYN1* variants. Seizures onset ranges from six months to 26 years of life in individuals with *SYN1* variants, with a median age of onset of seven years. For those patients for whom seizures are reported, seizures manifest for the first time before the second year of life in 29% of the cases, and within the first five years of life in 55% of the cases. Before the 13th year of age, 90% of the individuals has had the first seizure.
